# Spatio Temporal EEG Source Imaging with the Hierarchical Bayesian Elastic Net and Elitist Lasso Models

**DOI:** 10.3389/fnins.2017.00635

**Published:** 2017-11-16

**Authors:** Deirel Paz-Linares, Mayrim Vega-Hernández, Pedro A. Rojas-López, Pedro A. Valdés-Hernández, Eduardo Martínez-Montes, Pedro A. Valdés-Sosa

**Affiliations:** ^1^The Clinical Hospital of Chengdu Brain Science Institute, MOE Key Lab for Neuroinformation, University of Electronic Science and Technology of China, Chengdu, China; ^2^Neuroinformatics Department, Cuban Neuroscience Center, Havana, Cuba; ^3^Department of Biomedical Engineering, Florida International University, Miami, FL, United States; ^4^Politecnico di Torino, Turin, Italy

**Keywords:** EEG source imaging, inverse problem, sparsity regularization, sparse Bayesian learning, empirical Bayes, elastic net, mixed norms, elitist lasso

## Abstract

The estimation of EEG generating sources constitutes an Inverse Problem (IP) in Neuroscience. This is an ill-posed problem due to the non-uniqueness of the solution and regularization or prior information is needed to undertake Electrophysiology Source Imaging. Structured Sparsity priors can be attained through combinations of (L1 norm-based) and (L2 norm-based) constraints such as the Elastic Net (ENET) and Elitist Lasso (ELASSO) models. The former model is used to find solutions with a small number of smooth nonzero patches, while the latter imposes different degrees of sparsity simultaneously along different dimensions of the spatio-temporal matrix solutions. Both models have been addressed within the penalized regression approach, where the regularization parameters are selected heuristically, leading usually to non-optimal and computationally expensive solutions. The existing Bayesian formulation of ENET allows hyperparameter learning, but using the computationally intensive Monte Carlo/Expectation Maximization methods, which makes impractical its application to the EEG IP. While the ELASSO have not been considered before into the Bayesian context. In this work, we attempt to solve the EEG IP using a Bayesian framework for ENET and ELASSO models. We propose a Structured Sparse Bayesian Learning algorithm based on combining the Empirical Bayes and the iterative coordinate descent procedures to estimate both the parameters and hyperparameters. Using realistic simulations and avoiding the inverse crime we illustrate that our methods are able to recover complicated source setups more accurately and with a more robust estimation of the hyperparameters and behavior under different sparsity scenarios than classical LORETA, ENET and LASSO Fusion solutions. We also solve the EEG IP using data from a visual attention experiment, finding more interpretable neurophysiological patterns with our methods. The Matlab codes used in this work, including Simulations, Methods, Quality Measures and Visualization Routines are freely available in a public website.

## Introduction

Electrophysiological Source Imaging (ESI) constitutes a relatively inexpensive and non-invasive approach to study neural activity with a high temporal resolution. ESI is a classic example of an Inverse Problem (usually referred to as EEG IP), given the little amount of data available as compared to the large number of parameters needed to model the spatially distributed whole brain activity this problem is ill-posed in the Hadamard sense (Hadamard, 1923). Its mathematical properties are in the first place determined by the forward model, i.e., the equation relating the electrical potential measured at the scalp (V) and the originating Primary Current Density (PCD) J created by the electrical activity of large neuronal masses in every time *t*, which is a Type I Fredholm Integral Equation^1^:

(1) $$V\left(r_{e},t\right)=\int K\left(r_{e},r\right)\cdot J\left(r,t\right)dr^{3}$$

Here, the kernel $K\left(r_{e},r\right)$ is called Electric Lead Field (ELF), and maps the space of brain generators *r* to the position of the measuring electrodes *r*_*e*_. The ELF can be computed by using the quasistatic approximation of Maxwell equations and a model of the geometric and electrical properties of the head (Riera and Fuentes, 1998). The EEG IP is then defined as estimating the parameters J given measurements V and a known ELF. In practice, discretization of Equation (1) leads to a linear system:

(2) V=KJ+ε

where the unknown parameters (*J*) constitute an *S* × *T* matrix (*T* is the number of time instants and *S* is the number of spatial generators, i.e., voxels) that represents the discretized PCD. *V* and ε are *N* × *T* matrices, representing the measured EEG and the measurement noise in *N* electrodes (generally no more than 128), usually distributed according to a standard system (Klem et al., 1999). It need be noted that the discretized ELF, *K*, is an *N* × *S* ill-conditioned matrix due to the large correlations among its columns. Since the region of potential brain generators is usually of thousands of voxels, then *N* << *S* and the system of linear Equation (2) is highly underdetermined, i.e., it does not have a unique solution. This discretization and dimensionality reduction affects the accuracy of reconstructed solutions. Additionally, the forward model by itself consists in an oversimplification of the real generator space of EEG data and electromagnetic properties of the head. Therefore, some authors have suggested to test the inverse solutions with a different head model than the one used for simulations, in order to evaluate the methods in typical real data scenarios, when the individual MRI and head model is not available. This would allow the study to avoid committing the so-called “Inverse Crime” (Kaipio and Somersalo, 2004).

### Sparsity regularization with L1/L2 norms models: the elastic net and the elitist lasso

A well-known way to find a unique solution to the EEG IP is through Tikhonov Regularization (Tikhonov and Arsenin, 1977). It uses additional or prior information on the parameters, which is usually introduced in the form of mathematical constraints and it is closely related to Penalized Least Squares (PLS), which is generally expressed as:

(3) $$\hat{J}=argmin_{J}\left\{{\left\|V-KJ\right\|}_{2}^{2}+\lambda P\left(𝕃J\right)\right\}$$

The first term in the cost function in (3) is the residual of the model and the second summarizes the imposed constraints (penalty terms). The regularization parameter λ controls the relative weight of the penalty function *P*. The matrix 𝕃 adds information about the correlation structure of the parameter matrix. In the context of the EEG IP, it has taken the form of the identity matrix (independent PCD in each voxel) or the Laplacian operator (discrete second derivative) for requiring spatial smoothness of the PCD.

A well-known example of Tikhonov Regularization is the *Ridge* method (Hoerl and Kennard, 1970), where the penalty function is the L2 norm of the parameters, Table 1a. Particular cases of this method have given rise to several well-established inverse solutions such as Minimum-Norm, Weighted Minimum-Norm and Low Resolution Electromagnetic Tomography (LORETA), Table 1b. Their advantages and disadvantages have been thoroughly studied (Pascual-Marqui, 1999).

**Table 1:** Different models for Tikhonov Regularization and their corresponding penalty functions.

	**Model**	**Penalty function**
a)	Ridge	$||J|{|}_{2}^{2}$
b)	LORETA	$||LJ|{|}_{2}^{2}$
c)	LASSO	||*J*||_1_
d)	LASSO Fusion	||*LJ*||_1_
e)	MPLS	$\underset{k}{\sum }\lambda_{k}\psi_{k}\left(J\right)$
f)	Fused LASSO	$\lambda_{1}\overset{S-1}{\underset{i=1}{\sum }}||J_{i,\cdot }|{|}_{1}+\lambda_{2}\overset{S-1}{\underset{i=1}{\sum }}||J_{i+1,\cdot }-J_{i,\cdot }|{|}_{1}$
g)	ENET	λ_1_||*J*||_2_+λ_2_||*J*||_1_
h)	ENETL	λ_1_||*LJ*||_2_+λ_2_||*LJ*||_1_
i)	Smooth LASSO	λ_1_||*LJ*||_2_+λ_2_||*J*||_1_
j)	MXN	$||J|{|}_{p,q}={\left(\overset{T}{\underset{t=1}{\sum }}{\left(\overset{S}{\underset{i=1}{\sum }}|J_{i,t}{|}^{p}\right)}^{\frac{q}{p}}\right)}^{\frac{1}{q}}$
k)	GLASSO	$\overset{S}{\underset{s=1}{\sum }}{\left(\overset{T}{\underset{t=1}{\sum }}|J_{i,t}{|}^{2}\right)}^{\frac{1}{2}}$
l)	ELASSO	$\overset{T}{\underset{t=1}{\sum }}{\left(\overset{S}{\underset{i=1}{\sum }}|J_{i,t}|\right)}^{2}$

Sparse PCDs have also been searched through methods based on such as the Least Absolute Shrinkage Selection Operator (LASSO) (Tibshirani, 1996), Table 1c, and its version LASSO Fusion (Land and Friedman, 1996), Table 1d, which use the L1 norm as the penalty function.

In the last years, sparsity constraints based on L1/L2 norm have become very popular to achieve a high spatial resolution, in reconstructing and differentiating sources of brain activity, associated to different cerebral functions/states. This constraint can also fit better with physiological knowledge about brain activity in particular experimental and real-life situations. However, its mathematical treatment is not straightforward and strongly depends on the models and algorithmic approaches.

A special example consists in algorithms performing smooth and sparse estimations in separate steps (Liu et al., 2005; Palmero-Soler et al., 2007) to explicit combining models as the sum of L1 and L2 penalty functions while using iterative algorithms to solve it (Nagarajan et al., 2006; Valdés-Sosa et al., 2009; Tian and Li, 2011).

These L1/L2 models have been included in a framework called Multiple Penalized Least Squares (MPLS) (Valdés-Sosa et al., 2006; Vega-Hernández et al., 2008), Table 1e, which consists in a generalization of formula (3) to multiples penalty terms with their corresponding lambdas.

#### The elastic net in the context of multiple penalized least squares models

A very promising approach in the context of MPLS is based on a flexible model, combining L1/L2 norms, called the Elastic Net (ENET) (Zou and Hastie, 2005), Tables 1g,h. Solving this model is of particular interest for incorporating together the advantages of the L2 norm (Ridge) and L1 norm (LASSO) family models. Other particular examples of MPLS' are the Fused LASSO (Tibshirani et al., 2005), Table 1f, based only in the L1 norm, and the smooth LASSO (Hebiri, 2008), Table 1i, combining L1 and L2 norms.

For minimizing the PLS's (or its MPLS generalization) cost function in (3), considering models such as the Elastic Net, many algorithms exhibit advantages in the convergence time. Some Important examples are general modified Newton-Raphson algorithms, such as Local Quadratic Approximation (LQA) (Fan and Li, 2001) and Majorization-Minorization (MM) (Hunter and Li, 2005), without a considerable loss of speed in computations.

In a seemingly different approach combining L1/L2 norms, some authors have used the idea of structured sparse penalization based on *mixed-norms* (MXN) models (Kowalski and Torrésani, 2009a,b), Table 1j. In this context using the L1 norm of a vector whose elements are obtained as the L2 norms of other vectors is known as Group Lasso (GLASSO) (Yuan and Lin, 2006; Kowalski and Torrésani, 2009a,b), Table 1k. An important application of the GLASSO penalty is for example the Focal Vector Field reconstruction (Haufe et al., 2008) where sparsity is imposed on the amplitude of the PCD but keeping smoothness in the 3 spatial components (x, y, z) that defines the direction of this vector magnitude. The penalization function is then the L1 norm of the vector formed by the L2 norms of the PCD vector in each voxel.

#### The elitist lasso in the context of mixed-norm models

With the same goal, a model based on the L2 norm of a vector whose elements are obtained as the L1 norms of other vectors, has been called Elitist Lasso (ELASSO) (Kowalski and Torrésani, 2009a,b), Table 1l. This type of penalization was extended to the spatio-temporal context, consisting in the application of an L1 norm along the first dimension of the parameter matrix, and an L2 norm along the second dimension (Ou et al., 2009). This model presents similarities with respect to the MPLS models discussed above, particularly the ENET, therefore it is a suitable candidate among the MXN models for taking advantages of algorithmic solutions developed for those ones.

Although originally a second-order cone programming was used (Ou et al., 2009), it has been shown that these models for imposing structured sparsity can be estimated by a generalized shrinkage operator (Kowalski and Torrésani, 2009b). However, the regularization approach using MXN as a penalty function becomes a convex, non-differentiable, irrational, and non-separable (along columns or rows) optimization problem, which makes the inference process computationally very expensive.

More recently, efficient proximal operators and gradient-based based algorithms have been developed to compute a solution to the spatio-temporal EEG IP, by using special cases of mixed-norms (e.g., FISTA, Beck and Teboulle, 2009; Gramfort et al., 2012, 2013).

#### Limitations of the regularization approach

The estimated solutions using the algorithms discussed above, for the MPLS and MXN formulations of L1/L2 norms, become very sensitive to the set of constraints selected to regularize them and to the values of the regularization parameters. Within the state-of-the-art literature, usually the regularization parameters are selected ad hoc, or via heuristic information criteria, as Akaike Information Criteria (AIC), Bayesian Information Criteria (BIC) and Generalized Cross Validation (GCV). These procedures lead to compute the Inverse Solution for a large set of values of the regularization parameters, in order to evaluate the information criteria, thus being computationally expensive, in many cases non-optimal and usually leading to an inadequate balance of the constraints. Critical examples are the Multiple Shrinkage Problem of MPLS models, such as the ENET (Zou and Hastie, 2005; Vega-Hernández et al., 2008), and the selection of regularization parameters for the MXN models in Gramfort et al. (2012, 2013) and Gramfort et al. (2014).

### Bayesian inference and sparse bayesian learning

As dealing with the regularization parameters estimation in the Classical Inference approach constitutes an important limitation, other approaches have been proposed within the Bayesian framework (Schmidt et al., 1999; Trujillo-Barreto et al., 2004; Wipf et al., 2006). The Bayesian Inference have emerged as an alternative formulation, which is more general and can incorporate models, equivalent to the Tikhonov Regularization.

This framework addresses the EEG IP in terms of finding the posterior *pdf* of the parameters (*J*) using the Bayes rule:

(4) p(J|V,α,β)∝p(V|J,β)p(J|α)

Where *p*(*V*| *J*, β) is the likelihood, *p*(*J* |α) is the parameters' prior and (α, β) are hyperparameters. The prior plays a similar role as a penalty function in the classical framework. As a particular case, the *maximum a posteriori* estimate in this formalism leads to a fully equivalent solution to the optimization problem in Equation (3), when the likelihood is a Gaussian (with mean *KJ* and unit variance) and the prior has the following exponential form (using α = λ):

(5) $$p\left(J|\alpha \right)=\frac{1}{z}e^{-\alpha P\left(𝕃J\right)}$$

An important advantage of the Bayesian framework is that in a second level of inference (MacKay, 2003) one can estimate the hyperparameters of interest, typically variances and/or precisions of the likelihood and the priors. One way to achieve this is by using the Empirical Bayes procedure, where the aim is to maximize the posterior of hyperparameters:

(6) $$\left(\hat{\alpha },\hat{\beta }\right)=argmax_{\left(\alpha ,\beta \right)}p\left(\alpha ,\beta |V\right)$$

This approach has been widely developed in the last years, especially with sparse priors, which has been called Sparse Bayesian Learning (SBL). An important example is the Relevance Vector Machine (RVM) (Tipping, 2001; Schmolck and Everson, 2007), consisting in estimating sparse parameter vectors (with a Gaussian prior) while learning the hyperparameters (with a Gamma prior). Although theoretically Bayesian Learning methods are more robust for computing the inverse solution, fast and efficient inference algorithms have been currently developed only for simple models (Tipping and Faul, 2003). Bayesian formulations of the ENET (Li and Lin, 2010) or multiple variants of LASSO in the MPLS family (Kyung et al., 2010) have been developed previously, by using scale Gaussian mixtures (Andrews and Mallows, 1974). Although scale Gaussian mixtures constitute a considerable improvement for computing these models still involves the use of computationally intensive Expectation-Maximization (EM) and/or Monte Carlo (MC) algorithms, which are impractical for the EEG IP due its high dimensionality.

### Objectives

In this work we propose a new method based on an Empirical Bayes formalism to promote Structured Sparsity through two models based on mixtures of L1/L2 norms penalization functions (Laplace/Normal priors):

- The **Elastic Net** (ENET) (Zou and Hastie, 2005; Vega-Hernández et al., 2008).
- The **Elitist Lasso** (ELASSO) (Kowalski and Torrésani, 2009a,b).

Although we develop the theory for ENET and ELASSO models, the whole methodology can be generally applied to other types of Laplace/Normal priors. The algorithm can be considered as a Sparse Bayesian Learning method since it allows to estimate the regularization (hyper-) parameters that control sparsity and perform variable selection, while at the same time avoiding Monte Carlo estimations or EM type algorithms.

The aims of this study are to present the theory and an insightful validation of the methods as electrophysiological source localization methods with a large set of simulations and real EEG visual Event Related Potentials. We also compare the performance of these models with other well-established methods in the ERP synthetic data and discuss the theoretical and experimental differences. The Matlab codes used in this work, including Simulations, Methods, and Quality Measures are freely available upon request to the authors.

## Methods

### Hierarchical elastic net model

The Bayesian formulation of the ENET can be interpreted as a Laplace/Normal prior model^2^, with associated hyperparameters (α_1_, α_2_) that are inversely related to the variances of the Normal and Laplace *pdfs*:

(7) $$p\left(J|\alpha_{1},\alpha_{2}\;\right)=\frac{1}{z}e^{-\sum_{t}\left(\alpha_{1,t}{\left\|J_{\cdot ,t}\right\|}_{2}^{2}+\alpha_{2,t}{\left\|J_{\cdot ,t}\right\|}_{1}\right)}$$

Where α_1, *t*_ and α_2, *t*_ represents the *t*th element of the hyperparameters vectors (α_1_, α_2_), the notation *J*_·,*t*_ refers to the *t*th column of *J*, and *z* is the normalization constant.

Since the squared L2 norm and the L1 norm are separable as a sum of terms depending on the individual components, this prior introduces stochastic independence in the whole spatio-temporal map of the parameters, i.e., Equation (7) can be factorized over voxels and time points (indexed by *i* and *t*, respectively) with corresponding normalization constants *z*_*i, t*_:

(8) $$p\left(J|\alpha_{1},\alpha_{2}\;\right)=\prod_{t,i}\frac{1}{z_{i,t}}e^{-\alpha_{1,t}J_{i,t}^{2}-\alpha_{2,t}\left|J_{i,t}\right|}$$

This prior can be expressed as a scale Gaussian mixture (Andrews and Mallows, 1974), with a mixing *pdf* corresponding to a Truncated Gamma on the new hyperparameter γ. This formulation can be found in the Bayesian literature with different parametrizations (Kyung et al., 2010; Li and Lin, 2010). In our case, it is convenient to reorganize the variances and mixing distribution as detailed in the following Lemma.

**Lemma 1** [Proof in Appendix A (Supplementary Materials)]: Let *J*_*i, t*_ be the random variable distributing with pdf $\frac{1}{z_{i,t}}e^{-\alpha_{1,t}J_{i,t}^{2}}e^{-\alpha_{2,t}|J_{i,t}|}$, where *z*_*i, t*_ is a normalization constant. Then the following equality holds:

(9) $$\begin{array}{l} \frac{1}{z_{i,t}}e^{-\alpha_{1,t}J_{it}^{2}-\alpha_{2,t}\left|J_{i,t}\right|}=\\ \;\int_{0}^{+\infty }N\left(J_{i,t}|0,\Lambda_{i,t}\right)TGa\left(\gamma_{i,t}|\frac{1}{2},1,\left(k_{t},\infty \right)\;\right)d\gamma_{i,t} \end{array}$$

(10) $$\Lambda_{i,t}=\frac{1}{2\alpha_{1,t}}{\bar{\Lambda }}_{i,t},\;{\bar{\Lambda }}_{i,t}=\left(1-\frac{k_{t}}{\gamma_{i,t}}\right)$$

(11) $$k_{t}=\frac{\alpha_{2,t}^{2}}{4\alpha_{1,t}}$$

Where $TGa\left(\gamma_{i,t}|\frac{1}{2},1,\left(k_{t},\infty \right)\;\right)$ is the Truncated Gamma pdf, with lower truncation limit *k*_*t*_.

**Figure 1:**
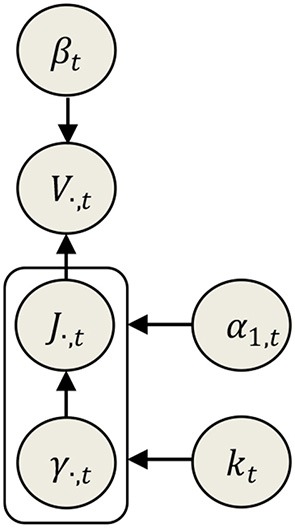
Directed network describing the instantaneous relationship between data (*V*_·,*t*_), parameters (*J*_·,*t*_) and hyperparameters (β_*t*_, γ_·,*t*_, α_1, *t*_, *k*_*t*_) in the Elastic Net model.

With this particular formulation of the ENET prior we change the relevant hyperparameters from the old regularization parameters (α_1_, α_2_) to (α_1_, *k*). The first one acts as the scale of variances of *J*_*i, t*_ (Equation 10), and the second acts as the lower truncation limit of the *pdf* for γ_*i, t*_. Although this makes more challenging the interpretation of the influence of the hyperparameters, it speeds the Empirical Bayes algorithm (described in section Structured SBL algorithm for the Elastic Net and Elitist Lasso models) and ensures its convergence. Given that *k* is now directly learned (without estimating α_2_, independently of α_1_), it is necessary to impose a convenient gamma prior for this hyperparameter. For α_1_ we can just choose a non-informative prior. Applied to the EEG IP stated in Equation (2), this new Bayesian ENET model can be analytically described by the following set of *pdfs*, see also the schematic representation in Figure 1.

Likelihood:

(12) $$V_{\cdot ,t}\sim N\left(V_{\cdot ,t}|KJ_{\cdot ,t},\beta_{t}I\right)$$

Prior of parameters:

(13) $$J_{\cdot ,t}\sim N\left(J_{\cdot ,t}|0,diag\left(\Lambda_{\cdot ,t}\right)\right)$$

Prior of hyperparameters:

(14) $$\gamma_{\cdot ,t}\sim \prod_{i}TGa\left(\gamma_{i,t}|\frac{1}{2},1,\left(k_{t},\infty \right)\;\right)$$

(15) $$\beta_{t}\sim \;non\text{-}informative,\;\log p(\beta_{t})=const$$

(16) $$\alpha_{1,t}\sim \;non\text{-}informative,\;\log p(\alpha_{1,t})=const$$

(17) $$k_{t}\sim Ga\left(\tau +1,\upsilon \right),\;\tau >0,\;\upsilon >0$$

Here β_*t*_ plays the role of the noise variance and τ and υ are the scale and shape parameters of the Gamma prior for *k*_*t*_.

In the original Bayesian ENET model the parameter's prior (8) controls the degree of sparsity/smoothness through variable selection according to the values of α_1_ and α_2_ directly, i.e., when α_1_ ≪ α_2_ we have that *p*(*J*|α_1_, α_2_) ≈ *La*(*J*|0, α_2_), promoting sparse solutions, and when α_2_ ≪ α_1_ we have that $p\left(J|\alpha_{1},\alpha_{2}\;\right)\approx N\left(J|0,\frac{1}{2\alpha_{1}}\;\right)$, which promotes smoothness for not-too-large values of α_1_. In our formulation of the ENET model the variable selection is performed in a similar fashion in the hyperparameters level, but in this case when *k* is large (α_1_ ≪ α_2_ for finite α_1_) we have by Equation (14) that γ ≈ *k*, which implies by Equation (10) that Λ → 0, finally promoting sparsity; and when *k* is small (α_2_ ≪ α_1_), $\Lambda \to \frac{1}{2\alpha_{1}}$ by Equation (10) so that in Equation (13) we have that $p\left(J|\gamma ,\alpha_{1},k\right)\approx N\left(J|0,\frac{1}{2\alpha_{1}}\right)$.

### Hierarchical elitist lasso model

Here, we propose a Bayesian formulation of the Elitist Lasso model using the following multivariate *pdf* :

(18) $$p\left(J\right)=\frac{1}{Z}e^{-\alpha {\left\|J\right\|}_{1,2}^{2}}$$

where α is a hyperparameter that controls simultaneously the level of spatial and temporal sparsity. This prior can be easily written for each time point separately by decomposing the mixed-norm as $||J|{|}_{1,2}^{2}=\underset{t}{\sum }||J_{\cdot ,t}|{|}_{1}^{2}$. However, the penalization over one spatial component *J*_*i, t*_ cannot be separated from the remaining components *J*_*j, t*_ (*j*≠*i*). We then propose to rearrange $||J_{\cdot ,t}|{|}_{1}^{2}$ in the form ${\left(\delta_{i,t}+|J_{i,t}|\right)}^{2}$, for any index *i*, where $\delta_{i,t}=\underset{j\neq i}{\sum }|J_{j,t}|$ is a magnitude carrying the dependence on the other components of the vector parameters. This allows us to derive a hierarchical Bayesian model for this formulation based on the theory of Markov Random Fields.

**Definition 1**: Let *x* be a random vector or random matrix, which has joint pdf of the form $p\left(x\right)=\left(\frac{1}{Z}\right)e^{-P\left(x\right)}$, where P(x) can be decomposed as $\underset{\left(i,j\right)\in J}{\sum }P_{ij}\left(x_{i},x_{j}\right)$; J is a set of pairs of index and *Z* is a normalization constant. Then we say that *x* is a “Pair-wise Markov Random Field” (pMRF), and the functions $\left\{P_{ij}\left(x_{i},x_{j}\right)\right\}$ are defined as potentials.

**Definition 2:** Let *x* be a random vector or random matrix. Let $x_{H}$ denote a subset of elements of *x*, which has conditional joint pdf of the form $p\left(x_{H}|x_{H^{C}}\right)=e^{-\alpha P\left(x\right)}/Z$, where $x_{H^{C}}$ is the complement of $x_{H}$ in *x*, P(x) can be decomposed as $\underset{\left(i,j\right)\in J}{\sum }P_{ij}\left(x_{i},x_{j}\right)$, $J\subseteq \left\{\left(i,j\right):x_{i}\vee x_{j}\in x_{H}\right\}$, and *Z* is a normalization constant. Then we say that $\left(x_{H},x_{H^{C}}\right)$ is a “Pair-wise Conditional Markov Random Field” (pCMRF). [Note that any couple of sets $\left(x_{H},x_{H^{C}}\right)$ from a pMRF constitutes a pCMRF; see the proof of Lemma 2 (a) in Appendix B (Supplementary Materials)].

In our case, the random vector consisting in a column of the spatio-temporal matrix *J* constitutes a pMRF, since its prior can be written as:

(19) $$p\left(J_{\cdot ,t}|\alpha \right)=\frac{1}{z}e^{-\alpha {\left\|J_{\cdot ,t}\right\|}_{1}^{2}}$$

Where the exponent can be decomposed as:

(20) $${\left\|J_{\cdot ,t}\right\|}_{1}^{2}=\sum_{i}J_{i,t}^{2}+\sum_{i>j}2\left|J_{i,t}\right|\left|J_{j,t}\right|\;;\;(i,j=1,\ldots ,S)$$

Hence the potentials of the Definition 1 are proportional to:

(21) $$P_{ij}=\{\begin{array}{c} J_{i,t}^{2}\;,i=j\\ 2\left|J_{i,t}\right|\left|J_{j,t}\right|\;,i\neq j \end{array}$$

pMRFs underlies many models of the Statistical Physics approach (Kindermann and Snell, 1980) and also learning algorithms (Murphy, 2012). In the case of the model described by Equations (19–21), we can reformulate the prior in a hierarchical model at the parameters level. For this, we need to use some relevant properties posed in the following Lemma.

**Lemma 2** [Proofs in Appendix B (Supplementary Materials)]: The following properties can be verified for the conditional probabilities in the pCMRF associated to the Elitist Lasso model of Equation (19).

(a)
  (22) $$p\left(J_{i,t}|J_{i^{C},t},\alpha \right)=p\left(J_{i,t}|\delta_{i,t},\alpha \right)=\frac{1}{Z_{i,t}}e^{-\alpha J_{i,t}^{2}}e^{-2\alpha \delta_{i,t}\left|J_{i,t}\right|}$$
  Where $\delta_{i,t}=\underset{j\neq i}{\sum }|J_{j,t}|$ and *Z*_*i, t*_ is a normalization constant.
(b)
  (23) $$p\left(\delta_{\cdot ,t}\right)=\frac{1}{\bar{Z}}e^{-\alpha {\left\|W^{-1}\delta_{\cdot ,t}\right\|}_{1}^{2}}I_{{ℛ}_{+}^{S}}\left(\delta_{\cdot ,t}\right)$$
  Where $I_{R_{+}^{S}}\left(\delta_{\cdot ,t}\right)$ is the indicator function of the region $R_{+}^{S}=\left\{x\in R^{S}:x_{i}\geq 0,i=1,\ldots ,S\right\}$, the matrix W can be expressed as the subtraction of the identity matrix from a ones-matrix: *W* = 1_*S* × *S*_ − 𝕀_*S* × *S*_ , and $\bar{Z}$ is a normalization constant.
(c)
  (24) $$\begin{array}{l} p\left(J_{i,t}|\alpha \right)=\int p\left(J_{\cdot ,t}|\alpha \right)dJ_{i^{C},t}=\\ \int \prod_{i}p\left(J_{i,t}|\delta_{i,t},\alpha \right)p\left(\delta_{\cdot ,t}|\alpha \right)dJ_{i^{C},t}d\delta_{\cdot ,t} \end{array}$$

**Figure 2:**
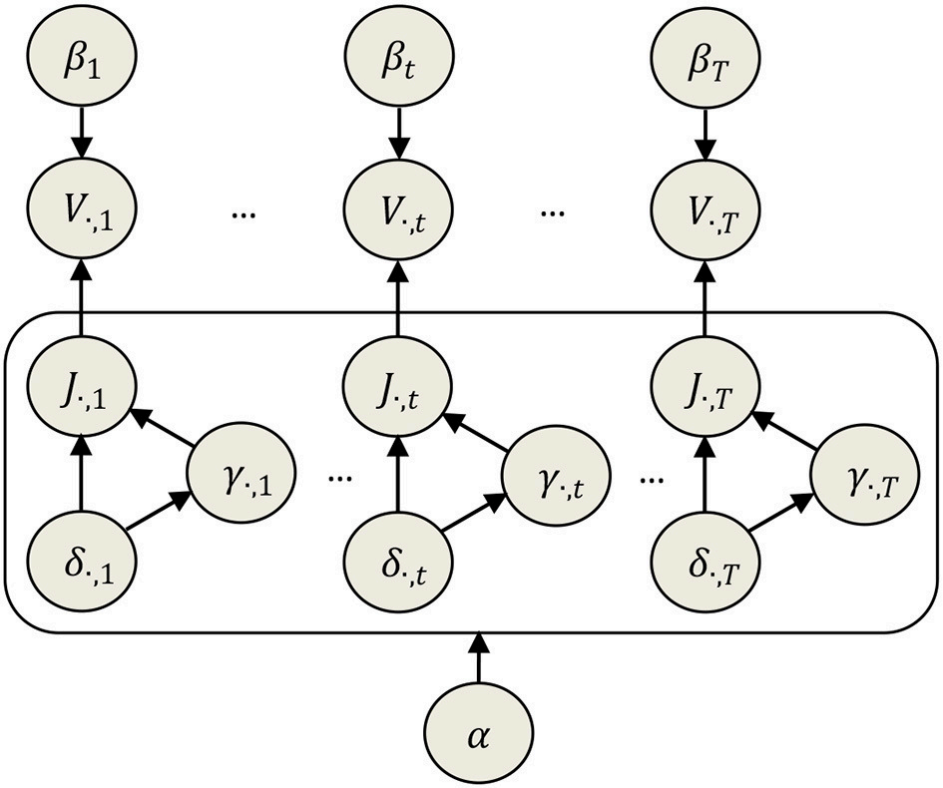
Directed network describing the instantaneous relationship between data (*V*), parameters (*J*), and hyperparameters (β, γ, δ, α) in the Elitist Lasso model. Note that alpha affects all time points simultaneously, i.e., it is just one hyperparameter controlling the same level of sparsity for all time points.

Using the properties (a,b) we show that taking the conditional *pdf* (22), instead of Equation (19), leads to a simpler model, separable in the parameters level. This implies considering δ_*i, t*_ as new hyperparameters, which inherit the multivariate behavior (spatial correlation structure) of *J* in the original model. As proved in Appendix B (Supplementary Materials), using a Dirac conditional distribution for this hyperparameters given the parameters, we can reformulate the non-separated model of Equation (19) into a hierarchical model that factorizes over different components of the parameters. Property (c) validates the use of the new model proposed in (a,b), showing that the marginal *pdf* of the parameters in the original model is identical to the marginal prior in the new one. Although this approximation is algebraically simpler, its effect might be related to other known approximations such as the mean field or a more general one in the Variational Bayes approach (Friston et al., 2007; Trujillo-Barreto et al., 2008). Also, it could be seen as a local ENET approximation of the ELASSO penalty, since the latter can then be explicitly re-written as a sum of an L2 and L1 norms of the parameters. Indeed, realizing that Equation (22) represents a combination of Normal and Laplace priors for *J*_*i, t*_, a further transformation of the Bayesian mixed-norm model is possible, combining the results in Lemma 1 and Lemma 2 and dealing with α and 2αδ_*i, t*_ as hyperparameters, similarly to α_1_ and α_2_ in the ENET model. The resulting prior of parameters is given below, the analogous schematic representation is showed in Figure 2:

(25) $$J_{\cdot ,t}\sim N\left(J_{\cdot ,t}|0,diag\left(\Lambda_{\cdot ,t}\right)\right)$$

(26) $$\Lambda_{i,t}=\frac{1}{2\alpha }{\bar{\Lambda }}_{i,t},\;{\bar{\Lambda }}_{i,t}=\left(1-\frac{\alpha \delta_{i,t}^{2}}{\gamma_{i,t}}\right)$$

And the priors of hyperparameters are:

(27) $$\gamma_{\cdot ,t}\sim TGa\left(\gamma_{i,t}|\frac{1}{2},1,\left(\alpha \delta_{i,t}^{2},\infty \right)\right)$$

(28) $$\delta_{\cdot ,t}\sim \frac{1}{Z}e^{-\alpha {\left\|W^{-1}\delta_{\cdot ,t}\right\|}_{1}^{2}}I_{{ℛ}_{+}^{S}}\left(\delta_{\cdot ,t}\right)$$

(29) $$\begin{array}{l} \beta_{t}\sim \;non\text{-}informative,\;\log p(\beta_{t})=const\\ \alpha \sim \;non\text{-}informative,\;\log p\left(\alpha \right)=const \end{array}$$

Similarly to ENET, the matrix *diag*(Λ_·,*t*_) can be interpreted as an effective prior variance of the parameters. However, here, the degree of sparsity in variable selection is twofold. On one hand, through the regularization parameter α, which is unique for the whole spatio-temporal map and imposes the same degree of sparsity to each column (time point) of parameters. On the other hand, the hyperparameters δ_*i, t*_ controls sparsity locally, so that when δ_*i, t*_ is large we will have $\gamma_{i,t}\approx \alpha \delta_{i,t}^{2}$, by Equation (27), and the effective prior variances will tend to zero Λ_*i, t*_ → 0, by Equation (26), promoting higher degree of sparsity independently of α.

### Structured SBL algorithm for the elastic net and elitist lasso models

For the Elastic Net and Elitist Lasso models the joint distribution of data, parameters, and hyperparameters in the spatio-temporal EEG IP admits the following factorization:

(30) $$p\left(V,J,\Theta \right)=\prod_{t}p\left(V_{\cdot ,t},J_{\cdot ,t},\Theta_{t}\right)$$

Where Θ represents the hyperparameters of both Elastic Net and Elitist Lasso: $\Theta_{t}=\{\begin{array}{c} \;\gamma_{\cdot ,t},\alpha_{1,t},k_{t},\beta_{t}\;;\text{ ENET}\\ \gamma_{\cdot ,t},\delta_{\cdot ,t},\alpha ,\beta_{t}\;;\text{ ELASSO} \end{array}\;$

Each factor in Equation (30) is the product of the likelihood, prior of parameters and priors of the hyperparameters:

$$p\left(V_{\cdot ,t},J_{\cdot ,t},\Theta_{t}\right)=N\left(V_{\cdot ,t}|KJ_{\cdot ,t},\beta_{t}I\right)N\left(J_{\cdot ,t}|0,diag\left(\Lambda_{\cdot ,t}\right)\right)$$

It is straightforward to derive an explicit formula for the maximum a posteriori estimate of the parameters *J*, from the data likelihood and its conjugated normal prior *pdf*, by using the result in formula [C1] of Appendix C (Supplementary Materials). The Empirical Bayes procedure then consists in minimizing the negative log-posterior of hyperparameters; see formula [C2] in Appendix C (Supplementary Materials).

(31) $$\begin{array}{l} L=-\log p\left(\Theta |V\right):\\ L=\frac{1}{2}\sum_{t}\{\log\left|\beta_{t}I\right|+\frac{1}{\beta_{t}}{\left\|V_{\cdot ,t}-K\mu_{\cdot ,t}\right\|}_{2}^{2}\;+\log\left|diag\left(\Lambda_{\cdot ,t}\right)\right|+\\ \;+\mu_{\cdot ,t}^{J}{\left(diag\left(\Lambda_{\cdot ,t}\right)\right)}^{-1}\mu_{\cdot ,t}\;+\log\left|{\bar{\Sigma }}_{t}^{-1}\right|-2\log p\left(\Theta_{t}\right)\} \end{array}$$

In Equation (31) μ_·,*t*_ and Σ_*t*_ represents the corresponding instantaneous parameter's posterior mean and covariance. It is important to note that these estimates are equivalent to the classical Minimum Norm Estimates in the regularization approach, when the hyperparameters are known or comprised into one hyperparameter that can be estimated through heuristic methods (e.g., minimizing information criteria or the generalized cross-validation function) (Hämäläinen and Ilmoniemi, 1994; Pascual-Marqui, 2002; Sánchez-Bornot et al., 2008; Vega-Hernández et al., 2008). However, here, with the Empirical Bayes approach, the estimation of hyperparameters is carried out by optimizing the posterior *pdf* of hyperparameters in Equation (31) (also known as Type II Likelihood). As hyperparameters control the level of sparsity, with an iterative algorithm, even simple models can lead to solutions totally different to the MNE solutions (MacKay, 1992, 2003; Schmidt et al., 1999; Friston et al., 2002, 2008; Trujillo-Barreto et al., 2004; Wipf et al., 2006).

The Type-II Likelihood in Equation (31) will take different forms depending on the model choice (ENET or ELASSO), since they use different expressions for the effective prior variances, according to Equations (10) and (26). In both cases, the hyperparameters' priors follow from Equations (14) to (17) and (27) to (29), respectively:

ENET:

$$\begin{array}{l} -\log p\left(\Theta_{t}\right)=\sum_{i}\left[\log\int_{k_{t}}^{\infty }Ga\left(x|\frac{1}{2},1\right)dx-\log Ga\left(\gamma_{i,t}|\frac{1}{2},1\right)\right]\\ \;-\log p\left(\alpha_{1,t}\right)-\log p\left(k_{t}\right)-\log p\left(\beta_{t}\right) \end{array}$$

ELASSO:

$$\begin{array}{l} -\log p\left(\Theta_{t}\right)=\sum_{i}\left[\log\int_{\alpha \delta_{i,t}^{2}}^{\infty }Ga\left(x|\frac{1}{2},1\right)dx-\log Ga\left(\gamma_{i,t}|\frac{1}{2},1\right)\right]\\ \;+\log\bar{Z}+\alpha {\left\|W^{-1}\delta_{\cdot ,t}\right\|}_{1}^{2}-\log p\left(\alpha \right)-\log p\left(\beta_{t}\right) \end{array}$$

To minimize the non-convex function L we use an iterative algorithm based on the coordinate descent strategy (Tipping, 2001; Wipf and Nagarajan, 2009), regarding the arguments $\left\{\mu ,\;\bar{\Lambda },\;\alpha ,\alpha_{1},\;k,\;\beta \right\}$, where we replaced γ by $\bar{\Lambda }$ in Equation (31) using Equations (10) and (26). We use the matrix Σ_*t*_, which is a non-linear function of the hyperparameters, for the estimation of parameters and hyperparameters in the next step. In the particular case of the mixed-norm we do not minimize L over the hyperparameter δ, due to the non-differentiability, but instead we just update it by using its relationship with parameters given in Lemma 2, (a). Appendix D (Supplementary Materials) shows the update formulas -and their derivations- based on the matrix derivative of L with respect to hyperparameters in each model.

Hereinafter, these methods for structured sparse Bayesian learning (SSBL) will be called ENET-SSBL and ELASSO-SSBL. The pseudo codes for the corresponding algorithms are given in Appendix F (Supplementary Materials). For implementation details and a further comprehension of our algorithms check Appendix E (Supplementary Materials).

## Results

### Simulation study and quality measures

**Figure 3:**
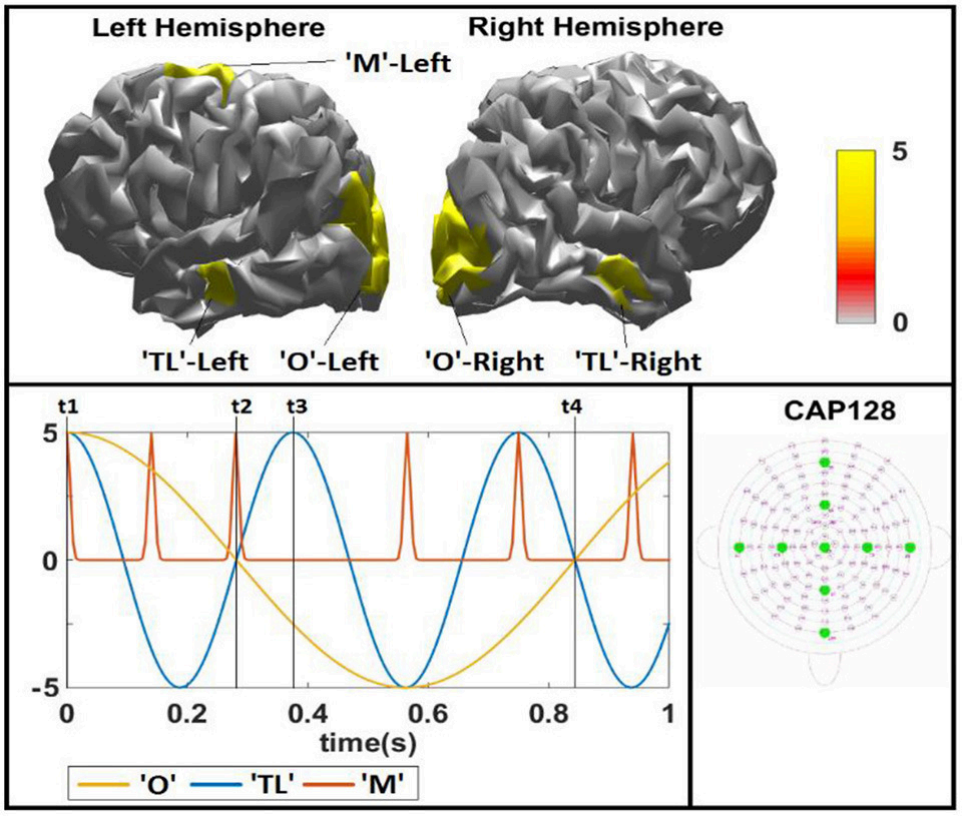
Simulated spatio-temporal sources. **Top:** Left and right views of the simulated activation patches in generators space (red). Five simulated patches with different geodesic spatial extension (Occipital “O” 30 mm, Temporal Lobe “TL” 20 mm, Motor “M” 10 mm) in the Left and only Occipital and Temporal Lobe in the Right hemisphere. **Bottom:** Left: Simulated time evolution of sources, for the “O” (~1 Hz cosine, yellow), the “TL” (~3 Hz cosine, blue), and the “M” (narrow Gaussian pulse signal). We highlight some time points of interest (t1 = 0 ms, t2 = 280 ms, t3 = 375 ms, t4 = 845 ms) where relative intensity of sources lead to qualitatively different scenarios. Right: Spatial representation of the electrodes in the plane according to the 120–130 system.

We evaluate the proposed methods by reconstructing the spatio-temporal PCD from simulated EEG data. We selected 3 sources, called Occipital “O,” Motor “M” and Temporal Lobe “TL” patches, with different spatial extension with regard to the geodesic distance in the surface: 30, 20, and 10 mm radius, respectively, as shown in Figure 3, top panel. The simulation was set up in a cortical surface of 6003 possible generators extracted from the MNI template brain (http://www.bic.mni.mcgill.ca). We generated 4 different spatial configurations using these patches to explore situations with different hemispherical distribution of the three sources: Simulation 1 with all sources in the left (L) hemisphere (O-L, M-L, TL-L); Simulation 2 where the occipital source is changed to the right (R) hemisphere (O-R, M-L, TL-L); Simulation 3 changing the TL source to the right (O-L, M-L, TL-R); and Simulation 4 moving both O and TL to the right hemisphere (O-R, M-L, TL-R). Time courses of 1 s, using a 200 Hz sampling frequency (201 time points), were also simulated for each patch, as shown in Figure 3, bottom panel. Time courses for patches O and TL were slow waves of 1 and 3 Hz, respectively. The frequency ratio (1/3 Hz) was chosen in order to reach enough variability along time in the relative intensity of these sources. The time course for patch M corresponded to a narrow Gaussian pulse that appears at six different time points when the other two patches showed qualitatively different relative intensity. This course became sparse after thresholding it to set all time points with intensity below 0.1% of the maximum equal to 0. In general, this set up of temporal courses (one sparse and two smooth in time) for each source allows not only to test the ability of the inverse methods to correctly estimate the time course of activations but also to model many different combinations of number and extension of simulated sources, i.e., their effective sparsity. Figure 3 shows that there are time points when all sources are active (e.g., *t1* = 0 ms), others where only one (e.g., *t2* = 280 ms) or two (e.g., *t3* = 375 ms) are active or even those when none of them are active (e.g., around 845 ms). Different amplitudes in the time courses imply different amplitudes of corresponding active voxels in each source, effectively changing the extension of each source and the general sparsity of the map. With this type of simulation, we can explore the behavior of the performance of inverse solutions, and their estimated hyperparameters with the different levels of sparsity, directly by looking at them along time points. Note that this can also be considered a challenging non-ideal scenario for the methods proposed here, given the presence simulations with simultaneous sources of different extensions, also following different temporal courses.

The ELF was computed for 120 sensors disposed on the scalp (selection of Biosemi 128 electrodes cap), using the BEM method for the individual head model (volume conduction), with 4 layers: scalp, outer skull, inner skull, gray matter (Valdés-Hernández et al., 2009). The spatio-temporal scalp electric potential (EEG) was computed as the product of the ELF and the simulated PCD, corrupted by adding biological noise in the sources, following an autoregressive process with spectral peak between 8 and 12 Hz (Alpha rhythm). We also add sensors noise as a Gaussian white process, such that both sources and electrodes noise were adjusted to obtained a signal-to-noise ratio of 6 dB. We generated 100 trials for each of the 4 simulated spatio-temporal sources by adding 100 samples of the biological and sensors noise in order to avoid a biased interpretation of the results due to a good choice of noise by chance. To avoid the “Inverse Crime” which recognizes that usually EEG source localization is done using standard (approximate) electrodes positions, head geometry, tissue conductivities, etc., instead of the real individual head model, some authors have proposed to use an ELF with decreased spatial resolution of the generators space for computing the solutions (Kaipio and Somersalo, 2004). Remarkably, we use a more challenging approach consisting in computing the solutions using an ELF obtained from a different subject (i.e., changing the locations of the generators corresponding to similar anatomical areas and also the geometry of the whole head model).

For comparison purposes, we computed the LORETA, ENETL and LASSO Fusion solutions using classical regularization approach (Hunter and Li, 2005; Sánchez-Bornot et al., 2008; Vega-Hernández et al., 2008). Computations were done by using the Elastic Net linear regression software package GLMNET (Qian et al., 2013; https://web.stanford.edu/~hastie/glmnet_matlab/). The regularization parameters for these three methods were computed as those minimizing the GCV function in a suitable interval made up of 100 values. All these inverse solutions used a graph Laplacian matrix, which introduces prior information about neighbors' structure in the generators space. All codes used in this work, including simulations, inverse solutions, quality measures and visualization of the results are freely available upon request to the authors.

For a quantitative evaluation of the solutions, we computed some quality measures based on the comparison with the true simulated one. There are two distances: the Dipole Localization Error, DLE, which measure how far are the estimated local maxima from the simulated ones and the more general Earth Mover's Distance between simulated and estimated solutions (Grova et al., 2006; Haufe et al., 2008; Molins et al., 2008). The lower these measures are, the better the match between estimated and simulated PCD. Two other performance measures offer were derived from a Receiver Operating Characteristic (ROC) analysis: the “area under the curve” (AUC), which is a measure of how good is the estimated solution for resembling the simulated one using any threshold, and the source retrieval index (F1-score) which measures a compromise between the fraction of simulated sources correctly estimated as active (recall or sensitivity) and the fraction of estimated sources that were simulated as active (precision) (Bradley et al., 2016; Hansen, 2017). Importantly, we did not threshold the estimated solutions for computing any of the distance measures. ROC analysis finds the AUC using the binarized simulated solution (nonzero sources will be set to 1) and the non-thresholded solutions. None of the inverse methods tested here incorporates any thresholding, thus making all voxels to have nonzero values. In order to compute the F1-score, we allow the ROC analysis to select the optimal operating point of the ROC curve (using *perfcurve* function in Matlab) and use the corresponding threshold to compute the recall (sensitivity) and precision. F1-score is the geometric mean between these two measures. This means that the methods will be compared according to their best performance individually. Here, we report AUC and F1 as percentages where the highest 100% values are achieved only by a perfect reconstruction of the simulated sources.

#### Bayesian elastic net and elitist lasso solutions

**Figure 4:**
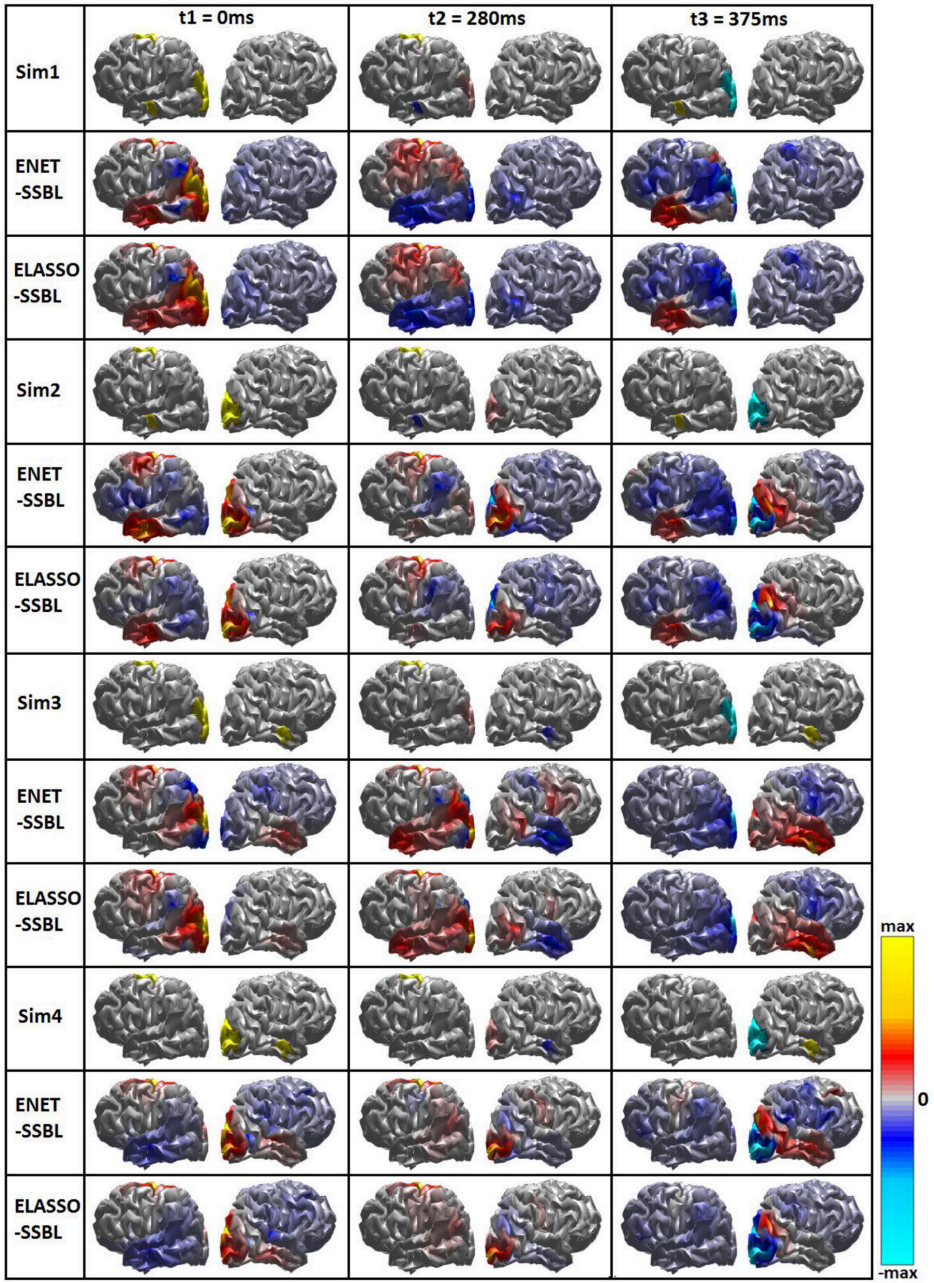
Simulated and estimated spatial maps with SSBL algorithm for the two proposed models ENET and ELASSO. Inverse solutions for a typical trial of each of the 4 simulated configurations (Sim1, Sim2, Sim3, and Sim4) at three time points with different sparsity. Bipolar color map shows the solution within a window of ± the maximum absolute value, gray color corresponds to zero values. A logarithmic scale has been set for a better visualization.

The inverse solution was computed with the ENET-SSBL and ELASSO-SSBL algorithms for the 100 trials of each simulated configuration. A typical trial was selected as the one ranking in the place 50 (median) after ordering all trials according to their best overall performance using all quality measures (sum of all rankings). Figure 4 shows the simulated and estimated spatial maps by both methods for the selected typical trials of each simulation, in the three time points of interest: *t1*, when all sources are similarly active; *t2* when the M source is maximum and the other two are close to zero and *t3*, when only the largest (O) and the smallest (TL) patches were nonzero.

**Figure 5:**
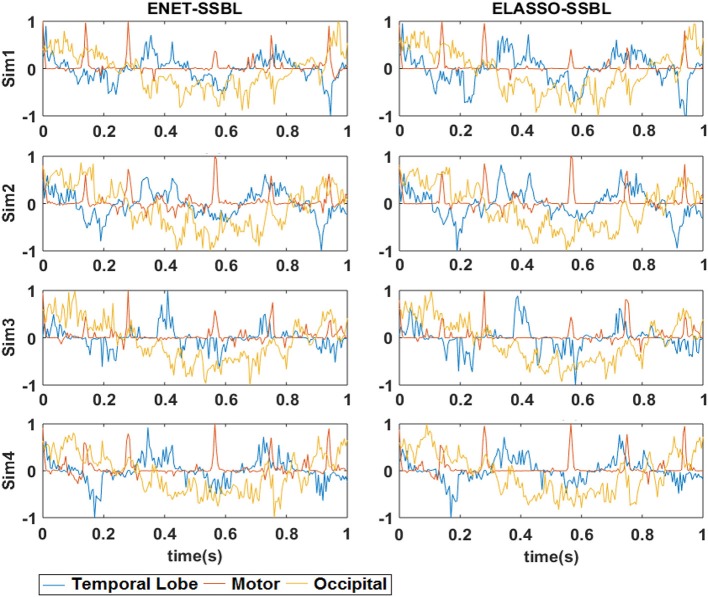
Estimated temporal courses with SSBL algorithm for the two proposed models ENET and ELASSO. The curves show the normalized temporal courses of the average activation across all voxels belonging to each of the three simulated spatial patches: Occipital (yellow), Temporal Lobe (blue), and Motor (red). These correspond to the same typical trials as Figure 4, for each of the 4 simulated configurations (Sim1, Sim2, Sim3, and Sim4).

Figure 5 shows the time evolution of the average activation of those voxels belonging to each patch, from both ENET-SSBL and ELASSO-SSBL solutions obtained in each simulated configuration (rows). These time courses are normalized between −1 and 1 for better visualization. Note that, despite being one trial in the median of the performance distribution, they resemble the original simulated time courses but without achieving the same level of smoothness for TL and O, or sparsity for M.

Finally, we computed the quality measures for each solution obtained using the two proposed methods from each of the 100 trials of each spatio-temporal simulation. Table 2 shows the average and standard deviation values across the 100 trials for all measures in the three time points of interest. We highlighted in bold the better values for each comparison. It is easy to see that even if it looks that ELASSO-SSBL gives better values in the majority of comparisons, the mean values of the measures are closer than the sum of their standard deviations, suggesting that both methods have similar performance according to these measures.

**Table 2:** Mean and standard deviation of the quality measures Earth Mover's Distance (EMD), Distance Localization Error (DLE), Area Under receiver operating Curve (AUC), and F1-Score (F1), across 100 trials, at time points t1 = 0 ms, t2 = 280 ms, and t3 = 375 ms of Simulations 1, 2, 3, and 4, for both algorithms ENET-SSBL and ELASSO-SSBL.

		**Simulation 1**		**Simulation 2**		**Simulation 3**		**Simulation 4**	
		**ENET-SSBL**	**ELASSO-SSBL**	**ENET-SSBL**	**ELASSO-SSBL**	**ENET-SSBL**	**ELASSO-SSBL**	**ENET-SSBL**	**ELASSO-SSBL**
EMD	t1	176 ± 50.1	**170.6** ± **38**	156 ± 58.1	**105.3** ± **49.1**	**186.8** ± **46.5**	192 ± 40.3	138.8 ± 54.2	**90.9 ± 26.1**
	t2	**124.1** ± **42.7**	125.2 ± 46.9	136.4 ± 69.2	**110.7 ± 52.7**	**122.8** ± **55.1**	124.5 ± 55.8	136.8 ± 73.9	**111.6** ± **45.8**
	t3	143.6 ± 55.9	**142.2** ± **56.3**	146.9 ± 68.6	**118.6** ± **52**	**143.6** ± **55.8**	149.3 ± 57.1	153.6 ± 82.2	**117.7 ± 61**
DLE	t1	1.77 ± 0.43	**1.48 ± 0.42**	2.78 ± 0.6	**2.73** ± **0.71**	2.06 ± 0.78	**2.03** ± **0.86**	2.81 ± 0.64	**3.24** ± **0.59**
	t2	3.03 ± 0.86	**2.92 ± 0.93**	3.77 ± 0.97	**3.76** ± **1.09**	3.45 ± 0.98	**3.19** ± **0.89**	4.2 ± 1.09	**4.13** ± **1.09**
	t3	2.09 ± 0.99	**1.9 ± 1.12**	3.21 ± 1.08	**2.83** ± **1.34**	2.57 ± 1.32	**2.41** ± **1.32**	3.96 ± 1.39	**3.85** ± **1.69**
AUC	t1	93.9 ± 1	**94.1 ± 0.7**	91 ± 1.4	**91.4** ± **1.2**	92.8 ± 1.4	**93.1** ± **1.4**	91.5 ± 1.1	**91.4** ± **1.3**
	t2	90.1 ± 2.9	**90.2 ±3.5**	**86.6** ± **5.6**	85.8 ± 5.9	88.6 ± 4.1	**89** ± **3.5**	85.9 ± 4.2	**85.3** ± **4.9**
	t3	93.7 ± 4.3	**93.9 ± 3.9**	**90.2** ± **4.5**	89.9 ± 6.1	93.6 ± 3.6	**93.7** ± **4.3**	90.7 ± 3.8	**90.6** ± **5.4**
F1	t1	61.5 ± 10.2	**67.6 ± 7.3**	35.2 ± 14.2	**38.1** ± **14.1**	60.7 ± 9.8	**67.3** ± **6.5**	37.3 ± 14	**40.2** ± **12.3**
	t2	37.8 ± 16.8	**40 ± 19.4**	**28.3** ± **17.5**	25.4 ± 18	37.5 ± 17.5	**38.8** ± **20**	**26.2** ± **17.5**	24.4 ± 18.5
	t3	50.7 ± 20.1	**50.8** ± **25.1**	34.1 ± 18	**36.4** ± **19.5**	54 ± 22.7	**56.4 ± 23.8**	35.6 ± 17	**37.4** ± **17.6**

*Best results between ENET-SSBL and ELASSO-SSBL algorithms are highlighted in bold, while the best results among all simulations (along rows) are underlined*.

#### Comparison of loreta, elastic net, lasso fusion and SSBL solutions

**Figure 6:**
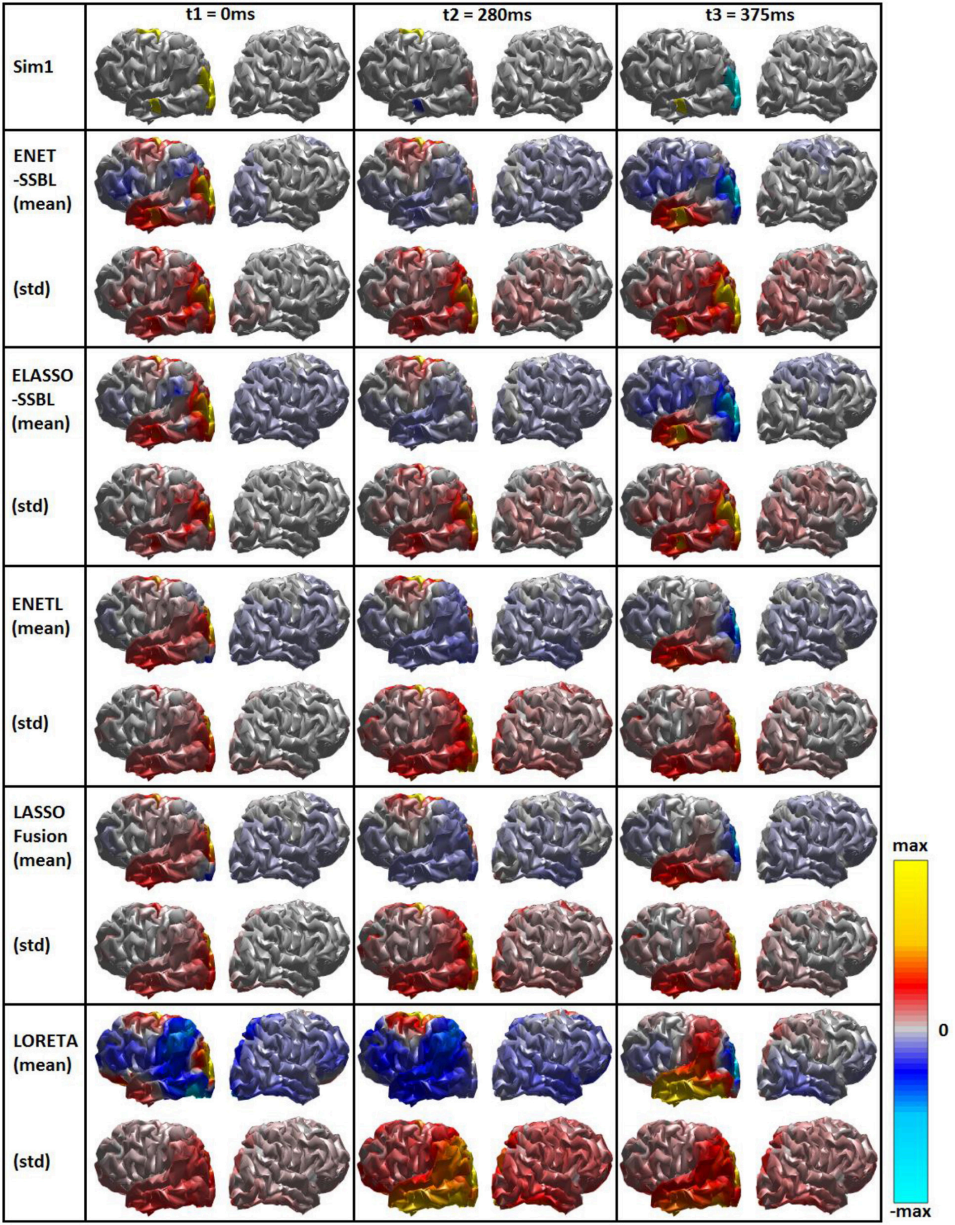
Simulated (first row) and estimated spatial maps with the different inverse methods. Each rectangle shows the mean (Upper part) and the standard deviation (Lower part) maps across 100 trials for the first simulated configuration (Sim1), at the three time points of interest corresponding to different sparsity scenarios. Bipolar color map shows the solution within a window of ± the maximum absolute value, gray color corresponds to zero values. A logarithmic scale has been set for a better visualization.

Figure 6 shows the simulated and estimated solutions using LORETA, ENETL, LASSO-Fusion, ENET-SSBL and ELASSO-SSBL in the same style as in Figure 4. In this case, the average and standard deviation maps across 100 trials are shown only for the first simulated configuration. The std maps are normalized by the maximum of the matching mean map, to better show those regions with high variability, which usually correspond to the regular appearance of spurious sources. In general, the proposed methods show the lowest variability and smaller regions of high variance -unrelated to true sources- than all other tested methods (especially in the sparsest *t2*).

**Figure 7:**
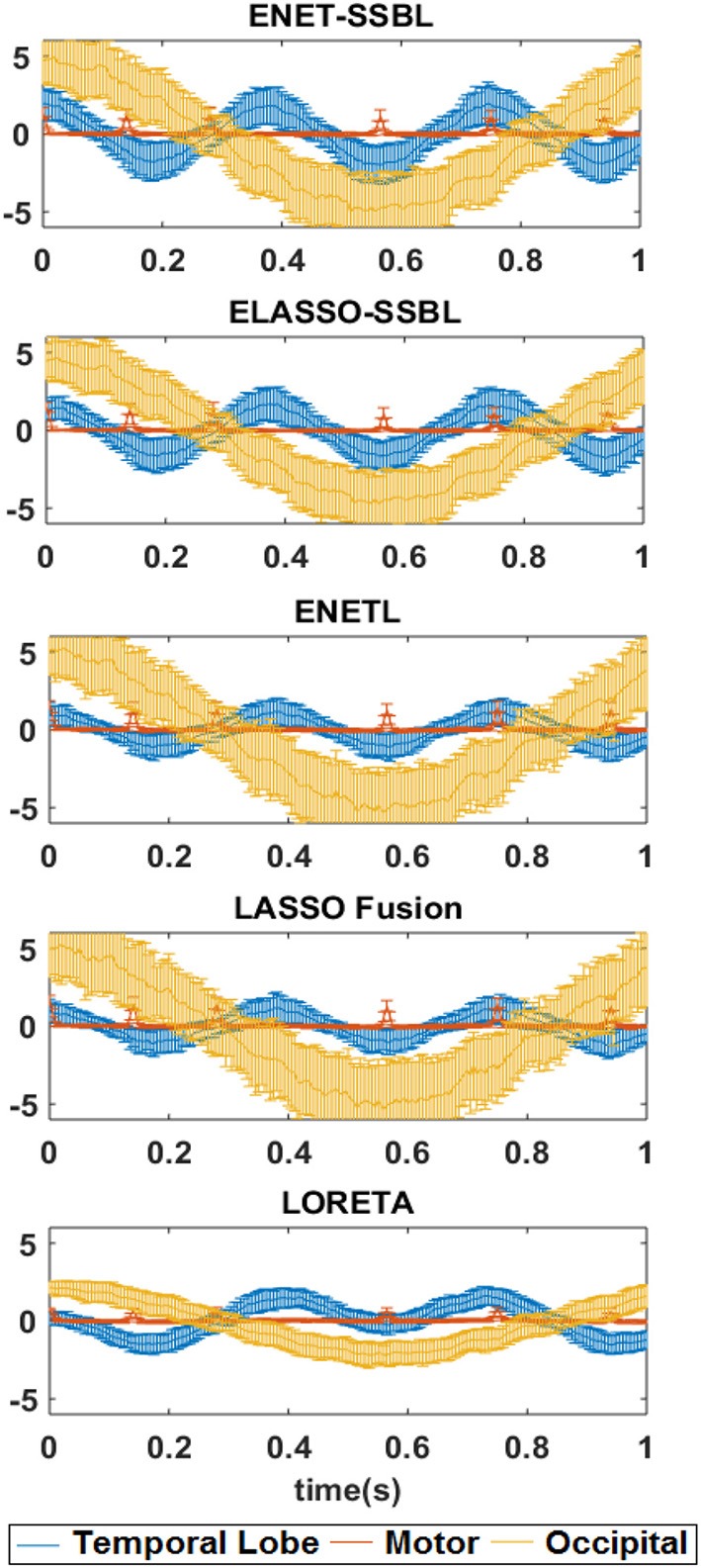
Estimated temporal courses with the different source localization methods for the first simulated configuration. The temporal courses correspond to the average activation across all voxels belonging to each of the three simulated spatial patches: Occipital (yellow), Temporal Lobe (blue) and Motor (red). The mean curves and standard deviation (error bars) across all trials are shown (without normalization).

Figure 7 shows the corresponding time evolution of the mean (curves) and std (error bars) of activations of voxels belonging to the different patches. In this case, we left the curves to show the scale of estimated solutions, in order to compare among different methods. Note that the estimation of the M patch is consistently biased with all methods, although the five narrow Gaussian pulses are clearly distinguished.

**Figure 8:**
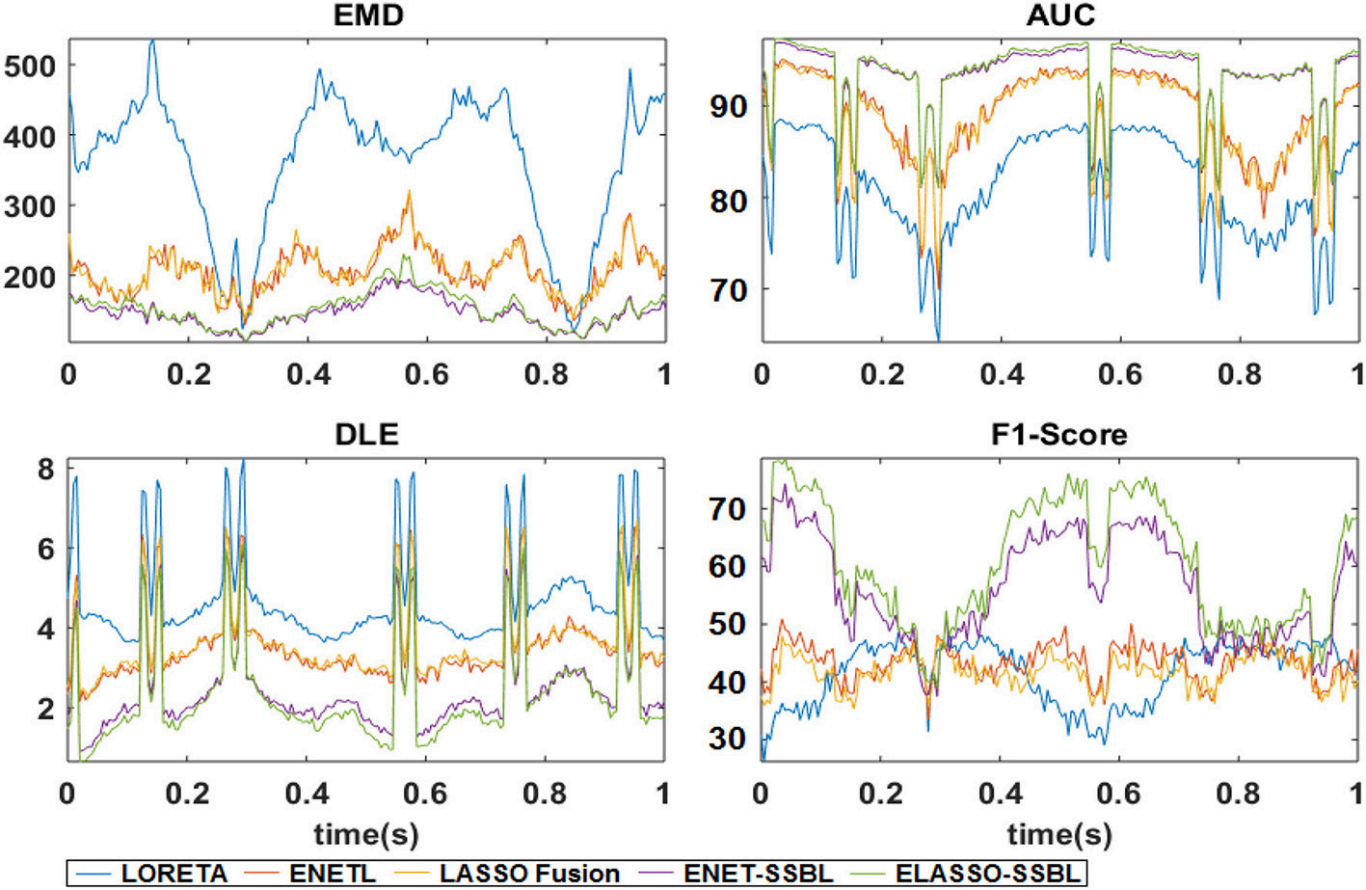
Mean curves along time of the quality measures Earth Mover's Distance (EMD), Distance Localization Error (DLE), area under receiver operating curve (AUC), and F1-Score, across 100 trials of Simulation 1 from estimated solutions with LORETA, ENETL, LASSO Fusion, ENET-SSBL, and ELASSO-SSBL.

Consequently, we computed the quality measures EMD, DLE, AUC and F1-Score for all trials of Simulation 1. Figure 8 shows the time evolution of the mean value of each quality measure for all methods. The sensitivity to the sparsity level in different time points is evident for all methods and all measures, especially showing the worst behavior around those time points where the M patch becomes active. However, it is clear that our two proposed methods offer the best average quality measures among all methods at all time points.

The mean and std values of all quality measures for the three time points *t1, t2* and *t3* in Simulation 1 are shown in Table 3A. Analogously to Table 2, we highlighted in bold the best performance for each row. In the average values, it is clearer that the ELASSO-SSBL achieves the best results overall, showing similar values as ENET-SSBL, but noticeably better than those of the other solutions. In order to test if these results were not driven by the particular selection of the spatial simulated patches, we ran 100 simulated trials with three similar patches but with randomized positions and extensions, without overlapping. Table 3B shows the corresponding mean and std values of the quality measures across these pseudo-random repetitions. It is easy to check that most of the values are very similar (within the std window) to those obtained in our particular simulated configuration, except for the F1-score which showed a much lower performance for all methods in this case. For each of these performance measures, we carried out non-parametric rank tests (Wilcoxon Rank-Sum Test, Gibbons and Chakraborti, 2011) between each pair of source localization methods, using all 100 trials of every time point of every of the four simulations. We then counted how many times (out of the 804 simulations, 201 time points × 4 simulations) each method was significantly better than another. Results are shown in Table 4. In this case, the two methods proposed here are almost always significantly better than any of the other methods in more than 50% of cases for all quality measures (except for the AUC comparison with ENETL).

**Table 3:** Mean and standard deviation across trials of the quality measures Earth Movers Distance (EMD), Distance Localization Error (DLE), Area Under receiver operating Curve (AUC), and F1-Score (F1) at the time points t1 = 0 ms, t2 = 280 ms, and t3 = 375 ms for all studied methods.

		**ENET-SSBL**	**ELASSO-SSBL**	**ENETL**	**LASSO Fusion**	**LORETA**
**(A) RESULTS ACROSS THE 100 TRIALS OF SIMULATION 1**						
EMD	t1	176 ± 50.1	**170.6** ± **38**	249.2 ± 139.2	259.2 ± 131.2	458.3 ± 86.8
	t2	**124.1** ± **42.7**	125.2 ± 46.9	165.8 ± 137.9	170.3 ± 124	252.8 ± 84.4
	t3	143.6 ± 55.9	**142.2** ± **56.3**	231.1 ± 183.3	235.4 ± 158.2	360.3 ± 144.8
DLE	t1	1.77 ± 0.43	**1.48** ± **0.42**	2.34 ± 0.73	2.51 ± 0.63	4.74 ± 0.81
	t2	3.03 ± 0.86	**2.92** ± **0.93**	3.67 ± 1.34	3.83 ± 1.32	4.9 ± 1.06
	t3	2.09 ± 0.99	**1.9** ± **1.12**	3.37 ± 1.2	3.37 ± 1.14	4.33 ± 1.08
AUC	t1	93.9 ± 1	**94.1** ± **0.7**	92.2 ± 2.1	91.4 ± 2	84.6 ± 2.8
	t2	90.1 ± 2.9	**90.2** ± **3.5**	85.3 ± 8	85.4 ± 7.7	74.5 ± 9.2
	t3	93.7 ± 4.3	**93.9** ± **3.9**	88.3 ± 9.9	88 ± 10.1	78.8 ± 12
F1	t1	61.5 ± 10.2	**67.6** ± **7.3**	42.2 ± 14.3	37.5 ± 14.1	30.8 ± 13.5
	t2	37.8 ± 16.8	**40** ± **19.4**	33.5 ± 18.5	36 ± 16.7	31.3 ± 20.9
	t3	50.7 ± 20.1	**50.8** ± **25.1**	42.4 ± 14.7	42.9 ± 14.3	46.8 ± 10.1
**(B) RESULTS ACROSS THE 100 TRIALS OF RANDOMIZED SIMULATION**						
EMD	t1	158.2 ± 72.7	**146.5** ± **54.5**	240.6 ± 139.3	239.5 ± 142.4	369.8 ± 133.8
	t2	116 ± 58	**109.8** ± **52.1**	161.6 ± 98.4	160.4 ± 95.4	207.4 ± 105.1
	t3	105.6 ± 61.8	**104.8** ± **58.2**	161.9 ± 101.4	167.1 ± 102.1	181.8 ± 82.6
DLE	t1	2.72 ± 1.23	**2.53** ± **1.17**	3.87 ± 1.63	4.07 ± 1.65	5.07 ± 2.21
	t2	4.12 ± 1.86	**3.93** ± **1.47**	5.61 ± 2.03	5.44 ± 2.03	6.24 ± 2.29
	t3	**3.52** ± **1.82**	3.53 ± 1.86	5.51 ± 2.72	5.65 ± 2.72	5.55 ± 3.33
AUC	t1	93.2 ± 4.4	**93.5** ± **4.3**	92.4 ± 4	92.3 ± 4	86.8 ± 6.8
	t2	86.7 ± 10.2	**87.8** ± **7.2**	83.7 ± 10.6	84 ± 10.8	77.1 ± 11.9
	t3	**92.5** ± **5.6**	92.4 ± 6	89.6 ± 10	89.9 ± 10.4	85.2 ± 14.3
F1	t1	48.2 ± 15.4	**49.4** ± **15.2**	45.9 ± 15.8	45.7 ± 15.8	45.8 ± 13.2
	t2	37.5 ± 16	36.8 ± 16.8	38.7 ± 17	39.9 ± 16.2	**42.8** ± **14.2**
	t3	44.1 ± 18.5	44.5 ± 18	46.6 ± 13.1	45.4 ± 14.5	**48.4** ± **13.9**

*Best results among algorithms (along rows) are highlighted in bold*.

**Table 4:** Statistical comparison of quality measures among all inverse methods, across all 100 trials at every time point for the 4 simulated configurations, using the non-parametric rank test.

	**ENET-SSBL**	**ELASSO-SSBL**	**ENETL**	**LASSO Fusion**	**LORETA**	**ENET-SSBL**	**ELASSO-SSBL**	**ENETL**	**LASSO Fusion**	**LORETA**
	**EMD**					**DLE**				
ENET-SSBL	–	2.1	69.8	69.7	90.2	–	1.5	87.1	88.9	97.5
ELASSO-SSBL	**49.8**	–	**77.61**	**79.9**	**92.2**	**39.7**	**–**	**90.4**	**91.7**	**99.3**
ENETL	2.6	0.1	–	0.1	85.3	1.9	0.4	–	4.6	86.7
LASSO Fusion	2.0	0.2	0.3	–	84.8	2.1	0.4	0	–	82.3
LORETA	1.4	0.1	0	0.1	–	0	0	0	0.124	–
	**AUC**					**F1-Score**				
ENET-SSBL	–	3.2	81.6	82.1	100	–	0	45.5	51.0	45.1
ELASSO-SSBL	**41.2**	–	**85.7**	**85.7**	**100**	**46.6**	**–**	**58.5**	**66.7**	**57.3**
ENETL	1.6	0.1	–	6.1	100	21.0	14.9	–	13.7	26.7
LASSO Fusion	1.1	0.2	0.2	–	100	14.2	9.0	0	–	19.2
LORETA	0	0	0	0	–	36.7	29.6	44.2	51.5	–

*Each cell shows the percentage in which the method in the row significantly outperforms the method in the column across all 804 (201 × 4) cases. Highest percentages along each column are highlighted in bold to show easily show the best algorithm*.

#### Estimated hyperparameters and sensitivity to sparsity levels

**Figure 9:**
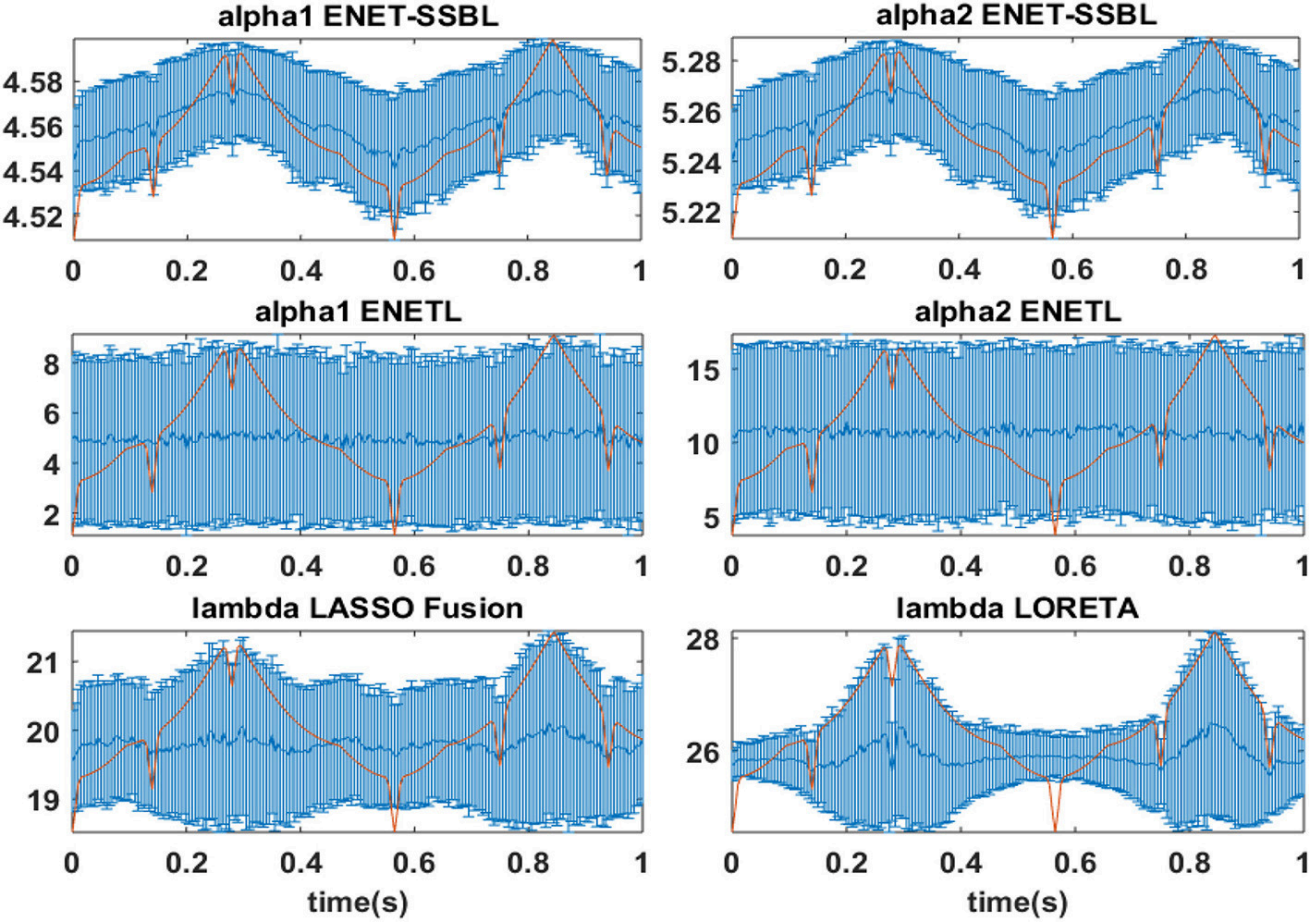
Hyperparameters estimates with the different source localization methods for the four simulated configuration. Blue curves represent the mean temporal behavior and standard deviation (error bars) across 400 trials of the logarithmic values of estimated hyperparameters with the different methods. These values were found with the ENET-SSBL algorithms and GLMNET toolbox by generalized cross validation. The red trace corresponds to the normalized sparsity level along time, i.e., the percentage of non-active voxels in the simulations.

One key point of this study is to test the ability of the proposed algorithm to correctly learn adequate values of the hyperparameters, and at the same time, check if the estimated hyperparameters effectively control the sparsity level of the solutions. In Figure 9 we show the time evolution of the average (curves) and standard deviation (error bars) of the logarithm of the two hyperparameters ENET-SSBL, the two regularization parameters of ENETL and the regularization parameters of Lasso Fussion and LORETA. The regularization parameters were found automatically by cross-validation performed by the GLMNET toolbox. It is interesting to see that only the learned hyperparameters of ENET-SSBL, which are optimized for each time separately, closely follows the level of sparsity of the simulated solution (red curve); LORETA shows changes of the variance of the parameter, but LASSO Fusion and ENETL give very similar values for all time points (i.e., different levels of sparsity). The level of sparsity was computed as 1 minus the L1 norm of the solution in each time point, normalized by the maximum value along time. For visualization purposes, the curves was adjusted to fit in the same range of the hyperparameters/regularization parameters shown in each panel. In the case of ELASSO-SSBL, the corresponding estimated values for the single hyperparameter was 5.18 ± 0.07. Typically, the SSBL algorithm for both methods reached convergence in around 15 iterations, 3.2 s (ENET-SSBL) and 4.2 s (ELASSO-SSBL) for obtaining a single inverse solution, in an Intel Core i5 CPU, at 1.70GHz and 4GB of RAM memory.

**Figure 10:**
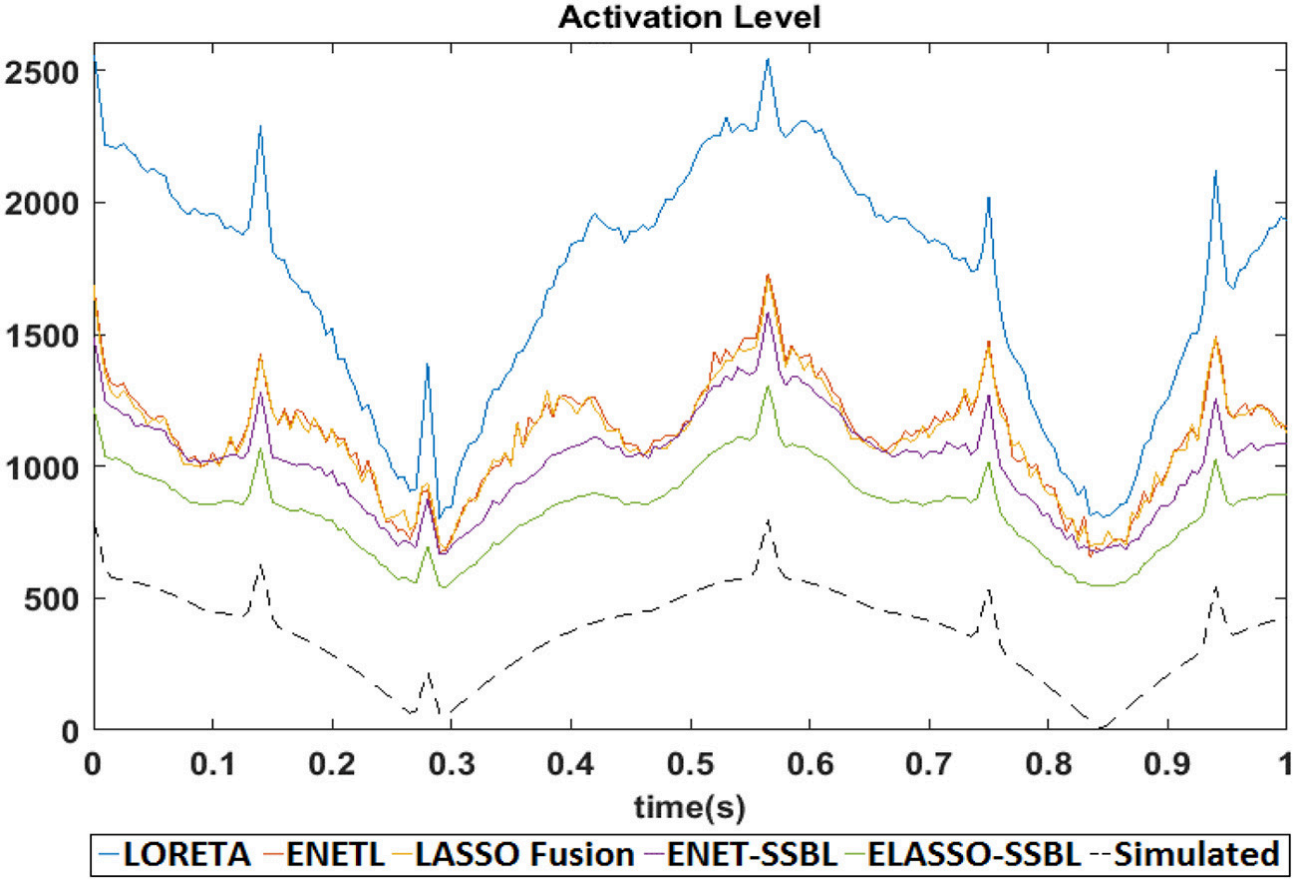
Temporal behavior of the average activation level (i.e., L1-norm) of simulated (dashed) and estimated solutions with the different methods. These curves are obtained from averaging all 100 trials of Simulation 1. We use the L1-norm as a measure of activation (since sparsity is related to its minimization), therefore those curves better resembling the curve of the simulated solution can be interpreted to represent solutions with a more similar level of sparsity to the sparsity of true sources.

We finally compute the real sparsity level of estimated solutions with the different methods and compare it with that of the simulated PCD in Simulation 1. Figure 10 shows the time evolution of the average L1 norms across the 100 trials of solutions for the different methods, together with that of the simulated solution (dashed curve). In this case, the L1 norm measures the level of activation, since it is usually minimized for sparse solutions, (i.e., it is an inverse measure of sparsity) and allows an easier relative comparison with the activation level of the simulated sources without normalization. Interestingly, all methods are able to resemble the change in sparsity of the simulation along time, although the ELASSO-SSBL solutions showed consistently closer values to the true ones among all other methods.

### Real data study of a visual attention experiment

**Figure 11:**
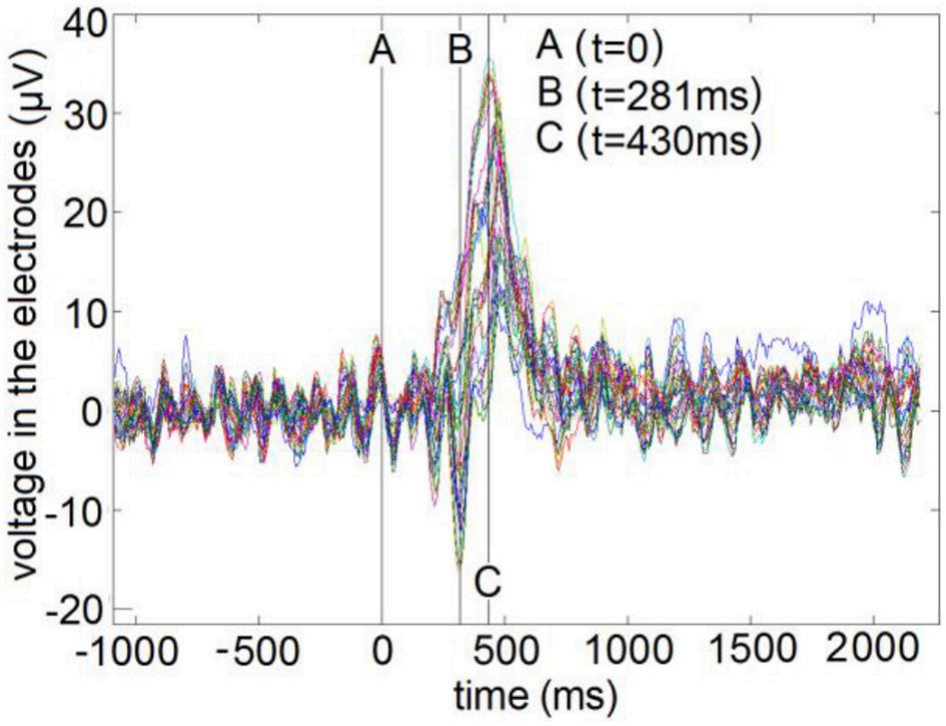
Time series of the Visual Evoked Potential for all electrodes. A, Reference time point (*t* = 0 ms) of stimulus onset. B, Global negative maximum potential (*t* = 281 ms). C, Global positive maximum potential (*t* = 430 ms).

EEG recordings using 30 electrodes (sampling frequency 128 Hz) were gathered from right-handed healthy subjects in a visual attention experiment devoted to study attentional modulation of early visual evoked potential, as explained in (Makeig et al., 1999, 2004; Makeig, 2002). Briefly, a sequence of geometric figures is presented as stimulus where the fixation point is defined as a cross and the subjects are requested to discriminate when a specific configuration appears in the attended location, by means of the physical action of pressing a button. The ethical clearance of this experiment was guaranteed by the Office of Naval Research according to the authors in the original paper (Makeig et al., 1999). The EEG recorded the brain activity for 25 to 75 repetitions of the stimulus, in 80 subjects, during a 3 s-long time window (1 s pre-stimulus and 2 s post-stimulus). Then, it is averaged over repetitions to cancel the background oscillatory activity. Figure 11 shows the 30 electrode's voltage time series (384 time points) of the Grand Average (average across subjects) Visual Evoked Potential, marking the stimulus onset (A) and the two time instants selected to compute the inverse solutions. These corresponded to the global maximum negative peak (B: 281 ms post-stimulus) and global maximum positive peak (C: 430 ms post-stimulus). The median reaction times (i.e., when the subjects pressed a button) was about 350 ms.

**Figure 12:**
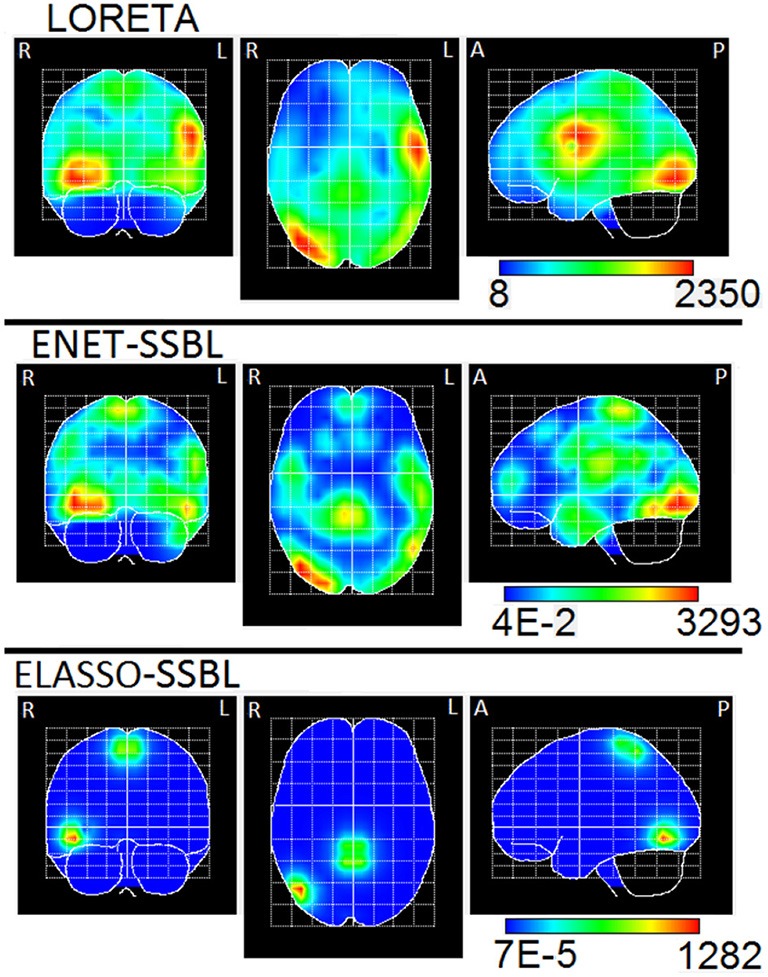
Maximum intensity projection of the PCD estimated with LORETA, ENET-SSBL, and ELASSO-SSBL, at the global negative maximum potential (time B in Figure 9). The three orthogonal planes are the coronal (left), axial (center) and sagittal (right) views. R, L, A, P stand for Right, Left, Anterior, Posterior, respectively.

**Figure 13:**
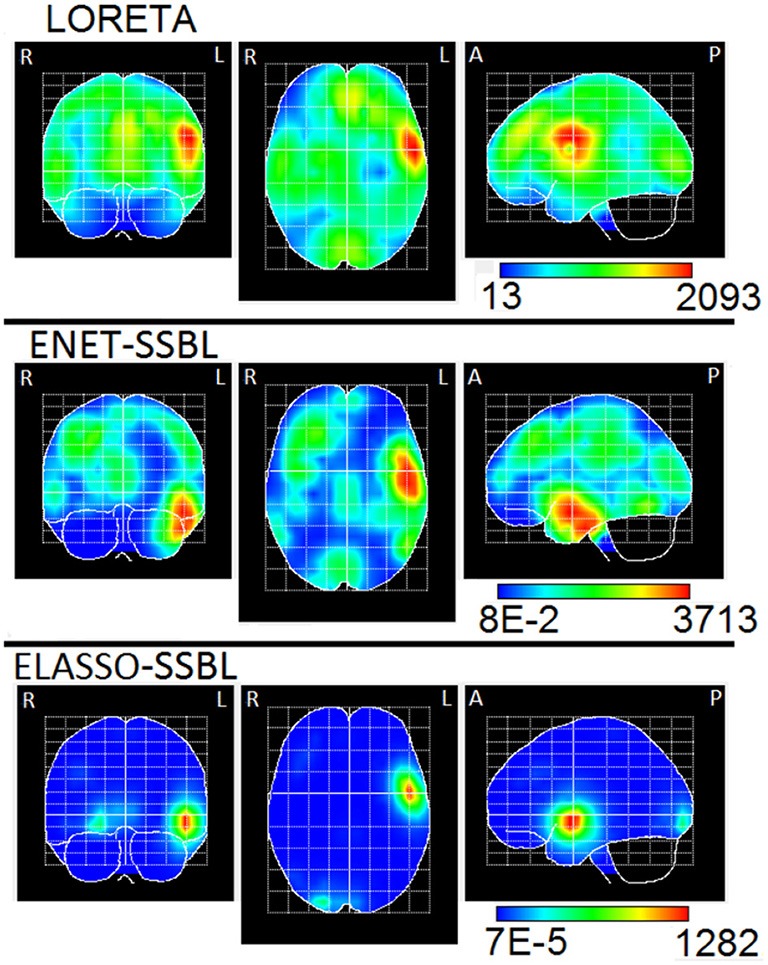
Maximum intensity projection of the PCD estimated with LORETA, ENET-SSBL, and ELASSO-SSBL, at the global positive maximum potential (time C in Figure 9). The three orthogonal planes are the coronal (left), axial (center) and sagittal (right) views. R, L, A, P stand for Right, Left, Anterior, Posterior, respectively.

To compute the ELF, we used an MNI standard brain and head model, defining a grid of 3244 generators (voxels) within the volume of the gray matter, the brainstem and the thalamus. The solutions were obtained by using the three orthogonal components of the Lead Field (three degrees of freedom), and also the same for the Laplacian matrix. The final sources maps were found as the L2 norm of each voxel of the estimated PCD. The ENET-SSBL and ELASSO-SSBL inverse solutions were compared with LORETA, one of the most used and well-studied solution. The results are visualized with the Neuronic Tomographic Viewer (http://www.neuronicsa.com/), using the maximum intensity projection view (i.e., projecting brain activations to three orthogonal planes). Figures 12, 13 show in color scale the solutions estimated with LORETA, ENET-SSBL and ELASSO-SSBL for the maximum negative and positive peak potentials (B and C time points, as marked in Figure 11), respectively.

**Figure 14:**
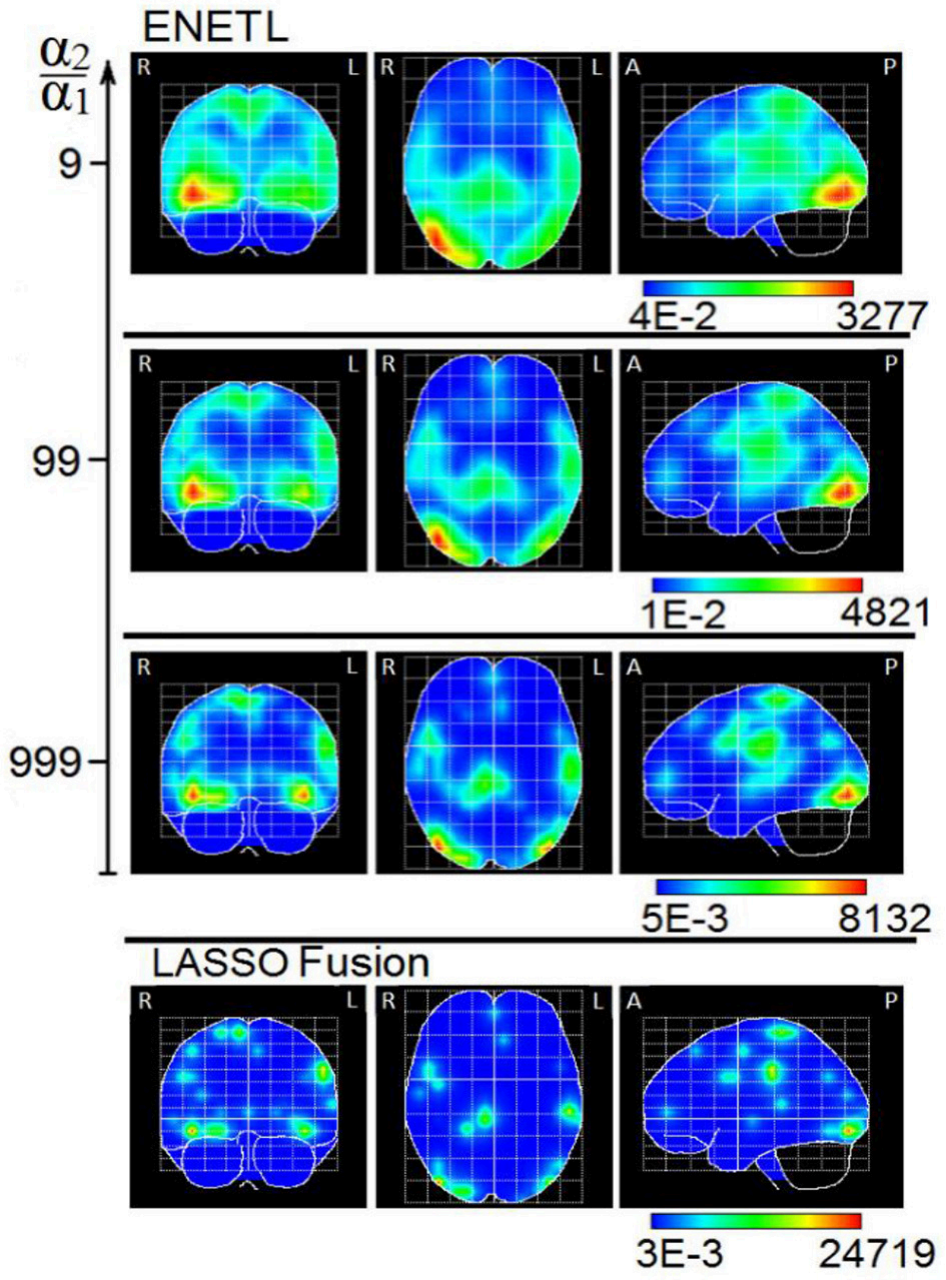
Maximum intensity projection of the PCD estimated with ENETL (using different ratio between regularization parameters) and LASSO Fusion at the global negative maximum potential (time B in Figure 9). The three orthogonal planes are the coronal (left), axial (center) and sagittal (right) views. R, L, A, P stand for Right, Left, Anterior, Posterior, respectively. This figure is a reproduction of results shown in Figure 3 in Vega-Hernández et al. (2008).

Solutions for the ENETL and for the LASSO Fusion models were also estimated with the MM algorithm. Figure 14 is a reproduction of Figure 3 in Vega-Hernández et al. (2008), and shows the ENETL solution with a set of regularization parameters, where λ is chosen via GCV for different fixed values of α (0.1; 0.01; 0.001). These values correspond to different ratios between regularization parameters (α_2_/α_1_ = (1 − α)/α) from 9 (low sparsity) to 999 (high sparsity).

## Discussion

### Analysis of the algorithms ENET-SSBL and ELASSO-SSBL

The Bayesian formulation of the ENET model proposed here is similar to Li and Lin (2010), and allows variable selection using the second level of inference for hyperparameters learning. Distinctively, we reformulate the hyperparameters as a scale factor (α_1_) of the parameters variances and the truncation coefficient (*k*) of the Truncated Gamma *pdf* that intervenes in variable selection. This allows the application of the Empirical Bayes procedure to estimate parameters and hyperparameters within an iterative coordinate descent algorithm, similar to the original RVM (Tipping, 2001; Wipf and Nagarajan, 2009). With this result, we avoid the Double Shrinkage Problem of the classical ENETL (Zou and Hastie, 2005; Vega-Hernández et al., 2008) and the use of computationally intensive MC/EM algorithms (Kyung et al., 2010; Li and Lin, 2010). Differently from the original RVM, here the hyperparameters (α_1_, *k*) controls the global degree of sparsity, while γ acts in variable selection over voxels individually. A possible extension of our algorithm could be derived from assuming different values of the hyperparameters over individual voxels or groups of voxels (region of interest), to impose a variable degree of spatial sparsity.

Our Bayesian formulation of the ELASSO model (Kowalski and Torrésani, 2009b) pursues a similar constraint of spatial sparsity and temporal smoothness in the parameters matrix as previous works (Haufe et al., 2008; Ou et al., 2009; Gramfort et al., 2012). However, this model represents a non-separable Markov Random Field in the spatial dimension of parameters (Kindermann and Snell, 1980; Murphy, 2012) that can be reorganized into a Normal/Laplace hierarchical model at the parameters level. This is done through the introduction of a new hyperparameter (δ) which implicitly impose the spatial correlation structure among the parameters. This approximation allows to perform variable selection and learning of the hyperparameters by means of Empirical Bayes in ELASSO model, which has not been previously reported in the literature. Interestingly, this approximation might be related to others that has been used in the Bayesian context -such as those in Variational Bayes and Mean Field approximation (Friston et al., 2007; Trujillo-Barreto et al., 2008)—while it could also be seen as a local ENET approximation of the ELASSO penalty. This interesting relationship and its possible algorithmic relevance should be explored in future studies.

One interesting theoretical result in this work is that both ENET and ELASSO models can be expressed as particular cases of combined Normal/Laplace priors. Therefore, our algorithmic approach might be useful for many other models (including Group LASSO, Smooth LASSO, combinations of ENET and ELASSO, and others). Some of them have been tackled in the Bayesian Approach, but only using MC/EM algorithms.

### Analysis of simulations

We tested the performance of the proposed methods in a simulated scenario where activations were not completely sparse in space nor completely smooth in time, thus challenging the assumptions of the models and exploring their capacity to adapt in non-ideal conditions. We also use a completely different brain and head model (based on the Lead Field of different subjects) for simulating the data and reconstructing the PCD, avoiding the inverse crime in more unfavorable conditions than the reported before in the literature. Finally, we have added noise in both the level of sources and sensors, as have been suggested in the literature (Haufe et al., 2013; Haufe and Ewald, 2016). As there is no ground truth for the EEG IP, we cannot know if this option is a more realistic situation or more challenging than typical adding of only sensors white noise. Similarly, it is not easy to use real EEG background signals as only “noise” (Kobayashi et al., 2003; Stephen et al., 2003), since it might carry information about other active sources that can obscure the important results about the ability to recover controlled real sources. In any case, using both sources of noise is not modeled directly by any of the methods studied here, thus becoming an additional challenge for the reconstruction. We also here always reported results obtained from 100 repetitions with different noise in every spatio-temporal configuration, for avoiding bias in interpretation due to a random “good” choice of the simulated noise.

In the analysis of our simulations, on one hand, ENET-SSBL was able to recover spatially patch-wise smooth solutions (Figures 4, 6), by accordingly tuning the degree of sparsity through the values of the hyperparameters, consistently with the theory (Figure 9). We also found a monotonous convergence pattern of the target likelihood function L for this solution in a single trial. The estimated values of hyperparameters were sensitive to the time-varying degree of sparsity, so that the values of the hyperparameters (α_1_ and α_2_) behaved consistently with the theory (Figure 9). They are higher when the degree of sparsity is higher. Noticeably, in 400 simulations (100 trials with SNR of 6dB for 4 different combinations of the three sources across hemispheres), the learning procedure of the proposed algorithm showed to be very robust, showing low standard deviation values in all time points. This is a very promising property -given that the EEG IP is also a very ill-conditioned problem- and it should thus be more thoroughly explored in future studies. On the other hand, the ELASSO-SSBL was also consistent with the theory, showing a monotonous convergence pattern. In Figure 4, the maps of ENET-SSBL and ELASSO-SSBL obtained for a typical trial (ranking in the median of the quality measures) are very similar, with the latter being slightly sparser in some of the simulations. The average time courses of the reconstructed sources in voxels belonging to simulated patches were similar to the original simulated ones, but much noisier (Figure 5). The occipital source seemed to be easier to recover, while the motor one was mostly absent, and it was very difficult to estimate its amplitude with either method.

The proposed methods were also compared in average with those estimated with the known methods LORETA, ENETL and LASSO Fusion (Hunter and Li, 2005; Sánchez-Bornot et al., 2008; Vega-Hernández et al., 2008). This was illustrated for the first simulated configuration, which is challenging as all three sources are active in the same hemisphere (Figure 6). LORETA showed the smoothest average maps, also with higher variance in all regions not involving real activations, which suggests that spurious sources are regularly shown by this solution. In the case of ENETL and LASSO Fusion, the solutions were able to recover the large patches (O and M) but missed the smaller patch “TL” in the left hemisphere. ENET-SSBL and ELASSO-SSBL did recover all sources better, although showing a higher variance except in the sparser situation occurring at *t2*. The non-normalized average time courses for the voxels belonging to the three simulated patches (Figure 7) showed again that the amplitude of the occipital patch (the largest in extension) is easier to recover, while the other two smaller patches are systematically underestimated by all methods. As expected, LORETA was the solution that offered the more biased estimation, although with the lowest variance in all patches.

A more general comparison in performance of the solutions was done using the quality measures in Figure 8, showing that ENET-SSBL and ELASSO-SSBL had smaller distances (EMD and DLE) and higher performance measures (AUC and F1-score) than ENETL, LASSO Fusion and LORETA. Quantitatively, ELASSO-SSBL and ENET-SSBL have similar results, but they both outperforms LORETA, ENETL, and LASSO Fusion, consistently along all different sparsity scenarios (Table 3). ELASSO-SSBL gives the best results for all time points in almost all measures (highlighted in bold in Table 3A). In the case of randomized simulations (Table 3B), LORETA showed the highest F1 score for those time points where only one or two sources were active with small amplitude. As these values are computed using the optimal threshold for each solution, this can be due to very low optimal thresholds that allow a very high recall for LORETA thanks to its smooth behavior (Bradley et al., 2016). However, these differences are not likely to be significant since the standard deviation of F1-score is very high. The significant non-parametric comparisons between all pair of methods according to all quality measures (Table 4) showed that ELASSO-SSBL is better than all other regularization methods in more than the 50% of all simulation cases. Particularly, DLE and AUC values of ELASSO-SSBL are significantly better than those of the other regularization methods (ENETL, LASSO Fusion, LORETA) in more than 85%, while it is significantly worst in less than 1% of the cases.

If we compare the two proposed models, we will find that in general ELASSO-SSBL performed slightly better than ENET-SSBL (Figure 8 and Tables 2, 3), which might be due to a better recover of the spatial extension of the simulated patches. However, this difference is significant in about 40% of the simulation cases (Table 4), which means that there are many scenarios where they give similar results. This might be interpreted as a higher performance of the spatio-temporal assumptions in ELASSO-SSBL, which is able to adapt to an adequate degree of sparsity by means of incorporating information of the spatial patches across all time points into the estimates of the regularization parameter.

Finally, computation times were similar in ENET-SSBL and ELASSO-SSBL, but the latter is more memory demanding because the estimation of the hyperparameter depends on parameters of the whole spatio-temporal map. Convergence was reached around 30 iterations of explicit estimators for a computational complexity of *O*(*N*^2^) (*N* is the number of electrodes). Although we did not use the MCMC algorithm for our simulations, other studies in the literature report that these methods take about 10 or 100 thousand iterations to converge, (Jun et al., 2005, 2008; Nummenmaa et al., 2007; Li and Lin, 2010). The cost of MCMC in the EEG Inverse Problem is determined by the Gibbs Sampler of the parameters posterior distribution, that amounts to *O*(*S*^2^) (*S* is the number of spatial generators).

The contrast between the superiority of ELASSO-SSBL in these general results (Table 4) and the less evident superiority in previous figures can be explained by the use of more challenging situations and time points for presenting the first results. That, together with the use of a completely different ELF to avoid the “inverse crime,” the use of both physiological and sensors noise sources and of simulations that do not follow the assumptions of our models, would suggest that the Empirical Bayes algorithm for the models proposed here is very robust to many of the typical factors that influence practical ESI, thus becoming a promising method to realistic experimental situations.

This robustness might be closely related to the efficacy in adequately estimating the hyperparameters of the model, which controls the levels of spatial sparsity and temporal smoothness. Figure 9 shows that the hyperparameters learned by ENET-SSBL closely follows the level of sparsity of the simulated solutions. The estimated regularization parameters with LORETA (Figure 9) also showed some correlation with the level of sparsity, especially for its variance, but smaller than the correlation showed by ENET-SSBL. Regularization parameters for ENETL and LASSO Fusion did not show sensitivity to the changes in sparsity, which could be explained by a wrong selection of the appropriate range for these parameters in the heuristic GCV procedure used for its estimation. Importantly, all methods showed in average a very similar pattern of the level of activation of their solutions to that of the simulated sources along time points (Figure 10). This would contradict the logical suggestion that a correct learning of the hyperparameters (i.e., following the sparsity of real sources) leads to estimate solutions with similar sparsity. In this case, we found that the level of activation for ENETL, LASSO Fusion and ENET-SSBL were similar in average. However, ELASSO-SSBL showed the smallest L1-norm of activated sources (highest degree of sparsity) along all-time points and it was also the closest to the real level of sparsity of the true simulation, resembling its shape along all time points. This result is very interesting as this method uses only one hyperparameter to control the level of sparsity for all time points simultaneously. As expected, LORETA showed the lowest sparsity along space/time, which usually conveys the cost of introducing spurious sources.

### Analysis of real data

In the analysis of real data, the maximum of the estimated solution with the three methods (LORETA, ENET-SSBL and ELASSO-SSBL) in the negative peak (time B in Figure 11), is located in the occipital area of the right hemisphere (Brodmann area 19), that corresponds to the visual cortex (Figure 12). As expected, LORETA is the most disperse solution, such that secondary activities appear not only in occipital areas but also in the superior temporal cortex. ENET-SSBL showed similar solutions than LORETA, but less disperse, such that the secondary activities in temporal, frontal and centro-parietal areas can be distinguished among them. Contrary to LORETA, the secondary activation in the temporal cortex was not the most intense, but the centro-parietal one in the motor cortex (Brodmann area 4). The solution with the ELASSO-SSBL method was the sparsest, showing only the main source in the right visual area and a secondary centro-parietal activation (motor cortex). Results from ENET-SSBL and ELASSO-SSBL are more consistent with the neurophysiology of visual attention where pre-motor activations in the occipital visual areas explain the processing of visual information, while secondary activations in the motor cortex are also expected related to the physical response (Hillyard and Anllo-Vento, 1998; Di Russo et al., 2001).

A great similarity between ENET-SSBL and ENETL solutions can be seen in the case when the ratio between regularization parameters in the latter is 99, while the hyperparameters are learned in the former (see Figures 12, 14). Although preliminary, this suggests that the learning strategy might be an effective way to find optimal intermediate levels of sparsity in real scenarios. It would also be very useful since learning the hyperparameter would offer a direct and justified way of estimating its optimal value without the need of using heuristic methods and heavy statistical post-processing as in Vega-Hernández et al. (2008). This is especially important in the case of ENET, since it contains two regularization parameters leading to a bi-dimensional search space and the corresponding high computational burden.

On the other hand, the ELASSO-SSBL solution distinguishes from the very sparse but scattered LASSO Fusion solution (Figure 14). Encouragingly, this suggests that with ELASSO-SSBL the degree of sparsity can be adapted to show a few small activated regions instead of many point sources, which is indeed more realistic in cognitive experiments.

The inverse solutions in the later positive peak (time C in Figure 11) showed similar patterns as those computed in the negative peak with occipital and parieto-temporal activations (Figure 13). Again, this is in agreement with research on the neurological foundations of the visual evoked potentials, showing that late positive potentials can be related to processing of other cognitive aspects of visual information and attention, as well as post-motor responses, whose sources are likely to be found in the superior anterior temporal lobe (Makeig et al., 1999, 2004; Bonner and Price, 2013). However, in this case the three methods did not agree in the location of the main source. Yet very spread, LORETA solution showed the maximum activity in the superior temporal cortex (Brodmann area 43) and secondary activations in occipital, frontal and parietal areas, being difficult to decide which is relevantly separated from the main activation. Differently, ENET-SSBL and ELASSO-SSBL found the maximum activity in the anterior temporal lobe (Brodmann area 21). ENET-SSBL showed again a similar solution as LORETA with a better trade-off between sparsity and smoothness, which would allow to identify different sources. The ELASSO-SSBL solution is the sparsest again, clearly separating two PCD sources. The maximum activity was located in the superior gyrus of the anterior temporal lobe and the secondary activation in the occipital area. Again, sources were not scattered focal activations but well-localized smooth patches of activations. It seems that the ELASSO-SSBL applied to just one time instant (i.e., without temporal information) does not behave simply like the LASSO model (Laplace prior), but it is able to find intermediate levels of spatial group sparsity. More comprehensive studies are needed to completely characterize the performance of this model with the proposed sparse Bayesian Learning algorithm.

## Conclusions

In this work we introduced a Bayesian formulation of Structured Sparsity regularization models combining L1/L2 norm constraints, for solving the EEG IP. In particular, we developed ENET and ELASSO models that have been previously addressed with the classical statistical framework, which presents practical limitations for selecting optimal values for one or more regularization parameters that are critical for correctly estimate solutions. We have used the Empirical Bayes approach for deriving a Sparse Bayesian Learning algorithm that allows both the estimation of parameters and the learning of hyperparameters by means of iterative algorithms. Using simulations, we found that our methods ENET-SSBL and ELASSO-SSBL are able to recover complex sources more accurately and with a more robust behavior under different sparsity scenarios than the classical ENETL, LASSO-Fusion and LORETA inverse solutions. Importantly, we have used a large set of simulations that do not cope with the theoretical assumptions of the models, and explore different sparsity scenarios. The quality measures used in this work were also found from the non-thresholded solutions to avoid the influence of arbitrary post-processing. Proper methods for a statistically founded thresholding and a more careful evaluation of the Bayesian estimator will be the subject of future developments with these models. In a real EEG study with a visual attention experiment, our methods localized electrical sources of early negative and late positive components of the Visual Evoked Potential that are more interpretable from the neurophysiological point of view, as compared with other known methods such as LORETA, ENETL and LASSO Fusion. Theoretically, the principles behind the proposed algorithms can be applied to other models based on combined Normal/Laplace priors and other types of inverse problems such as image recovery. Other possible extensions to deal with the vector PCD field (i.e., estimating the three spatial components in each voxel) or with other multidimensional data, such as time-frequency and space-time-frequency EEG tensors, should also be considered as new lines of research.

## Ethics statement

The data used in this paper was taken from Makeig et al. (1999). It is available at the website http://sccn.ucsd.edu/eeglab/download/eeglab_data.set

## Author contributions

DP-L: Mathematical formulation of the Elastic Net and Elitist Lasso probabilistic models. Empirical Bayes formulation and inference formulas. Derivation and implementation of the algorithms ENET-SSBL and ELASSO-SSBL. Simulations and Real Data study. Analysis and interpretation of the results. Writing the paper. MV-H: Review, mathematical analysis and implementation of the Elastic Net in the context of Tikhonov Regularization. Review and mathematical analysis of the Bayesian Elastic Net. PR-L: Computational issues of the ENET-SSBL and ELASSO-SSBL algorithms. PV-H: Building the substrate for specific subject EEG forward model, quantitative analysis of inverse solutions. PV-S: Introduction in our work the theory MPLS models and Sparsity Constraints. Advising in the theory of the EEG Inverse Problem and software platforms. Advising in the mathematical issues of the Elastic Net as an MPLS model in the EEG Inverse Problem. Providing Simulations and Real Data. Analysis and Discussion of the Results. EM-M: Review on the EEG IP theory. Advising on Sparsity Regularization techniques in the context of Tikhonov Regularization and Sparse Bayesian Learning. Guide in the mathematical issues of Bayesian models and Empirical Bayes. Proposal of the Bayesian Analysis of the Elitits Lasso. Guide in developing the algorithms ENET-SSBL and ELASSO-SSBL. Analysis of the Results.

### Conflict of interest statement

The authors declare that the research was conducted in the absence of any commercial or financial relationships that could be construed as a potential conflict of interest. The reviewer MM and handling Editor declared their shared affiliation.

## Footnotes

^1^For the mathematical notation used in this paper see Appendix G in Supplementary Materials.

^2^Note that this mixture of Laplacian and Gaussian distributions is not the Normal-Laplace distribution described in Reed (2006).
