# Supplementary material for: Spatio Temporal EEG Source Imaging with the Hierarchical Bayesian Elastic Net and Elitist Lasso Models
Source: Front Neurosci. 2017 Nov 16;11:635. doi: 10.3389/fnins.2017.00635 (PMC5696363; doi:10.3389/fnins.2017.00635)
Supplement: Supplementary file 1 [file Presentation1.pdf]

SUPPLEMENTAL MATERIAL

SPATIO TEMPORAL EEG SOURCE IMAGING WITH THE HIERARCHICAL BAYESIAN ELASTIC NET AND ELITIST LASSO MODELS

Deirel Paz-Linares(1,2), Mayrim Vega-Hernández(2), Pedro A. Rojas-López(1,2), Pedro A. Valdés-Hernández(2,3), Eduardo Martínez-Montes(2,4) and Pedro A. Valdés-Sosa(1,2)

(1) The Clinical Hospital of Chengdu Brain Science Institute, MOE Key Lab for Neuroinformation, University of Electronic Science and Technology of China, Chengdu, China
(2) Neuroinformatics Department, Cuban Neuroscience Center, Havana, Cuba
(3) Department of Biomedical Engineering, Florida International University, United States
(4) DISAT, Politecnico di Torino, Turing, Italy

APPENDICES

A. Proof of Lemma 1

The Normal/Laplace *pdf* in [2-3] can be rearranged as:

$$e^{-\alpha_1 J_{i,t}^2} e^{-\alpha_2 |J_{i,t}|} = \frac{2}{\alpha_2} e^{-\alpha_1 J_{i,t}^2} La\left(J_{i,t} \middle| \alpha_2\right)$$

Using the Gaussian scale mixture model for the Laplace *pdf* (Park and Casella, 2008):

$$La\left(J_{i,t} \middle| \alpha_2\right) = \int_0^{+\infty} N\left(J_{i,t} \middle| 0, x_{i,t}\right) \frac{\alpha_2^2}{2} e^{-\frac{\alpha_2^2}{2} x_{i,t}} dx_{i,t}$$

We obtain:

$$e^{-\alpha_1 J_{i,t}^2} e^{-\alpha_2 |J_{i,t}|} = \int_0^{+\infty} e^{-\alpha_1 J_{i,t}^2} \frac{\alpha_2}{\sqrt{2\pi x_{i,t}}} e^{-\frac{J_{i,t}^2}{2x_{i,t}}} e^{-\frac{\alpha_2^2}{2} x_{i,t}} dx_{i,t}$$

The term to the right can be rearranged by multiplying and dividing by $\sqrt{1 + 2\alpha_1 x_{i,t}}$ as:

$$\alpha_2 \int_0^{+\infty} \frac{1}{\sqrt{1+2\alpha_1 x_{i,t}}} \frac{1}{\sqrt{2\pi \frac{x_{i,t}}{1+2\alpha_1 x_{i,t}}}} e^{-\frac{J_{i,t}^2}{2\frac{x_{i,t}}{(1+2\alpha_1 x_{i,t})}}} e^{-\frac{\alpha_2^2}{2} x_{i,t}} dx_{i,t}$$

With the change of variables $\gamma_{i,t} = \frac{\alpha_2^2}{4\alpha_1}(1 + 2\alpha_1 x_{i,t})$ and $\Lambda_{i,t} = x_{i,t}/\left(1 + 2\alpha_1 x_{i,t}\right) = \frac{1}{2\alpha_1}\left(1 - \frac{\alpha_2^2}{4\alpha_1 \gamma_{i,t}}\right)$, we arrive at:

$$\sqrt{\frac{\alpha_2^2}{4\alpha_1}} e^{-\frac{\alpha_2^2}{4\alpha_1}} \int_{\frac{\alpha_2^2}{4\alpha_1}}^{+\infty} \frac{1}{\sqrt{2\pi\Lambda_{i,t}}} e^{-\frac{J_{i,t}^2}{2\Lambda_{i,t}}} \gamma_{i,t}^{-\frac{1}{2}} e^{-\gamma_{i,t}} d\gamma_{i,t} =$$

$$\int_{\frac{\alpha_2^2}{4\alpha_1}}^{+\infty} N\left(J_{i,t} \middle| 0, \Lambda_{i,t}\right) \sqrt{\frac{\alpha_2^2}{4\alpha_1}} e^{-\frac{\alpha_2^2}{4\alpha_1}} \gamma_{i,t}^{-\frac{1}{2}} e^{-\gamma_{i,t}} d\gamma_{i,t}$$

Then, using the definition for the Truncated Gamma (Gamma *pdf* truncated in the interval $\left(\frac{\alpha_2^2}{4\alpha_1}, \infty\right)$):

$$TGa\left(\gamma_{i,t} \middle| \frac{1}{2}, 1, \left(\frac{\alpha_2^2}{4\alpha_1}, \infty\right)\right) = \sqrt{\frac{\alpha_2^2}{4\alpha_1}} e^{-\frac{\alpha_2^2}{4\alpha_1}} \gamma_{i,t}^{-\frac{1}{2}} e^{-\gamma_{i,t}} I_{\left(\frac{\alpha_2^2}{4\alpha_1}, \infty\right)}\left(\gamma_{i,t}\right)$$

We demonstrate that the Normal/Laplace *pdf* can be represented as the following scale mixture of Gaussians:

$$e^{-\alpha_1 J_{i,t}^2} e^{-\alpha_2 |J_{i,t}|} =$$

$$\int_0^{+\infty} N\left(J_{i,t} \middle| 0, \Lambda_{i,t}\right) TGa\left(\gamma_{i,t} \middle| \frac{1}{2}, 1, \left(\frac{\alpha_2^2}{4\alpha_1}, \infty\right)\right) d\gamma_{i,t} \quad ■$$

B. Proof of Lemma 2

a) Setting $x_{\mathcal{H}} = J_{i,t}$ and $x_{\mathcal{H}^c} = J_{i^c,t}$ into Definition 1, the conditional probability of one variable (node) regarding their complement in the pMRF can be expressed as:

$$p\left(J_{i,t} \middle| J_{i^c,t}, \alpha\right) = p\left(J_{\cdot,t} \middle| \alpha\right) / p\left(J_{i^c,t} \middle| \alpha\right) \quad \text{[B-1]}$$

Using [2-14] and [2-15], the conditional *pdf* in [2-13] can be decomposed as:

$$p\left(J_{\cdot,t} \middle| \alpha\right) \propto e^{-\alpha P_{ii} - 2\alpha \sum_{j \neq i} P_{ij} - \alpha \sum_{l \neq i, j \neq i} P_{lj}} \quad \text{[B-2]}$$

Which can be marginalized over $J_{i,t}$ to obtain:

$$p\left(J_{i^c,t} \middle| \alpha\right) \propto e^{-\alpha \sum_{l \neq i, j \neq i} P_{lj}} \int e^{-\alpha P_{ii} - 2\alpha \sum_{j \neq i} P_{ij}} dJ_{i,t} \quad \text{[B-3]}$$

Substituting [B-2] and [B-3] in [B-1] we can find the potentials of Definition 2 for the duet $\left(J_{i,t}, J_{i^c,t}\right)$:

$$p\left(J_{i,t} \middle| J_{i^c,t}, \alpha\right) = \frac{e^{-\alpha P_{ii} - 2\alpha \sum_{j \neq i} P_{ij}}}{\int e^{-\alpha P_{ii} - 2\alpha \sum_{j \neq i} P_{ij}} dJ_{i,t}}$$

$$= \frac{1}{\mathcal{Z}_{i,t}} e^{-\alpha J_{i,t}^2} e^{-\alpha \sum_{j \neq i} 2 |J_{i,t}| |J_{j,t}|}$$

Where $\mathcal{Z}_{i,t}$ is a normalization constant. Then, using the auxiliary magnitude $\delta_{i,t} = \sum_{j \neq i} \left|J_{j,t}\right|$, we finally obtain:

$$p\left(J_{i,t} \middle| J_{i^c,t}, \alpha\right) = \frac{1}{\mathcal{Z}_i} e^{-\alpha J_{i,t}^2} e^{-2\alpha \delta_{i,t} |J_{i,t}|} \quad ■$$

b) The auxiliary matrix (δ) can be seen as new hyperparameters with a marginal *pdf* as:

$$p\left(\delta_{\cdot,t} \middle| \alpha\right) = \int p\left(\delta_{\cdot,t}, J_{\cdot,t} \middle| \alpha\right) dJ_{\cdot,t} = \int p\left(\delta_{\cdot,t} \middle| J_{\cdot,t}, \alpha\right) p\left(J_{\cdot,t} \middle| \alpha\right) dJ_{\cdot,t}$$

The conditional *pdf* $p\left(\delta_{\cdot,t} \middle| J_{\cdot,t}, \alpha\right)$ can be represented by means of the Dirac distribution Δ, using the expected value $\sum_{j \neq i} \left|J_{j,t}\right| = W \left|J_{\cdot,t}\right|$ (where $W_{S \times S}$ is defined as $W_{ii} = 0$ and $W_{ij} = 1$ for $i \neq j$):

$$p\left(\delta_{\cdot,t} \middle| J_{\cdot,t}, \alpha\right) = \Delta\left(\delta_{\cdot,t} - W\left|J_{\cdot,t}\right|\right) I_{\mathcal{R}_+^S}\left(\delta_{\cdot,t}\right)$$

We can use this and [2-13] to obtain:

$$p\left(\delta_{\cdot,t} \middle| \alpha\right) = \int \Delta\left(\delta_{\cdot,t} - W\left|J_{\cdot,t}\right|\right) I_{\mathcal{R}_+^S}\left(\delta_{\cdot,t}\right) \frac{1}{\mathcal{Z}} e^{-\alpha \|J_{\cdot,t}\|_1^2} dJ_{\cdot,t}$$

Taking into consideration the symmetry of the argument with respect to the origin, and rearranging the Dirac delta function we obtain:

$$p\left(\delta_{\cdot,t} \middle| \alpha\right) = \frac{1}{|W|\mathcal{Z}} I_{\mathcal{R}_+^S}\left(\delta_{\cdot,t}\right) \int \Delta\left(\left|J_{\cdot,t}\right| - W^{-1}\delta_{\cdot,t}\right) e^{-\alpha \|J_{\cdot,t}\|_1^2} dJ_{\cdot,t}$$

$$= \frac{1}{\overline{\mathcal{Z}}} e^{-\alpha \|W^{-1}\delta_{\cdot,t}\|_1^2} I_{\mathcal{R}_+^S}\left(\delta_{\cdot,t}\right)$$

where $\overline{\mathcal{Z}} = |W|\mathcal{Z}/2^S$ and $|W|$ is the determinant of W. ■

c) The joint pdf of parameters and new hyperparameters δ is:

$$p(J_{\cdot,t},\delta_{\cdot,t}|\alpha) = \prod_i p(J_{i,t}|\delta_{i,t},\alpha)\,p(\delta_{\cdot,t}|\alpha)$$

Using final results from previous items a) and b), it becomes:

$$p(J_{\cdot,t},\delta_{\cdot,t}|\alpha) =$$
$$\prod_i \frac{1}{\mathcal{Z}_{i,t}} e^{-\alpha J_{i,t}^2} e^{-2\alpha\delta_{i,t}|J_{i,t}|} \frac{1}{\overline{\overline{\mathcal{Z}}}} e^{-\alpha\|W^{-1}\delta_{\cdot,t}\|_1^2} I_{\mathcal{R}_+^S}(\delta_{\cdot,t})$$

Marginalizing over $J_{i^{\mathcal{C}},t}$:

$$\int p(J_{\cdot,t},\delta_{\cdot,t}|\alpha)dJ_{i^{\mathcal{C}},t} = \frac{1}{\overline{\overline{\mathcal{Z}}}} e^{-\alpha\|W^{-1}\delta_{\cdot,t}\|_1^2} I_{\mathcal{R}_+^S}(\delta_{\cdot,t}) \times$$

$$\frac{1}{\mathcal{Z}_{i,t}} e^{-\alpha J_{i,t}^2} e^{-2\alpha\delta_{i,t}|J_{i,t}|} \int \prod_{j\neq i}\left[\frac{1}{\mathcal{Z}_{j,t}} e^{-\alpha J_{j,t}^2} e^{-2\alpha\delta_{j,t}|J_{j,t}|}\right] dJ_{i^{\mathcal{C}},t} =$$
$$= \frac{1}{\overline{\overline{\mathcal{Z}}}} e^{-\alpha\|W^{-1}\delta_{\cdot,t}\|_1^2} I_{\mathcal{R}_+^S}(\delta_{\cdot,t}) \frac{1}{\mathcal{Z}_{i,t}} e^{-\alpha J_{i,t}^2} e^{-2\alpha\delta_{i,t}|J_{i,t}|}$$

And marginalizing over $\delta_{\cdot,t}$ using the change of variable $\delta_{\cdot,t} = W|J'_{\cdot,t}|$, we obtain:

$$\int p(J_{\cdot,t},\delta_{\cdot,t}|\alpha)dJ_{i^{\mathcal{C}},t}d\delta_{\cdot,t} =$$
$$\frac{|W|}{2^S\overline{\overline{\mathcal{Z}}}} \int \frac{1}{\mathcal{Z}_{i,t}} e^{-\alpha J_{i,t}^2 - 2\alpha\left(\sum_{j\neq i}|J'_{j,t}|\right)|J_{i,t}| - \alpha\|J'_{\cdot,t}\|_1^2} dJ'_{\cdot,t}$$

Where we recall that $\overline{\overline{\mathcal{Z}}} = |W|\mathcal{Z}/2^S$. Inserting now the decomposition:

$$\|J'_{\cdot,t}\|_1^2 = {J'_{i,t}}^2 + 2\left(\sum_{j\neq i}|J'_{j,t}|\right)|J'_{i,t}| + \left(\sum_{j\neq i}|J'_{j,t}|\right)^2$$

And rearranging terms, we arrive at:

$$\int p(J_{\cdot,t},\delta_{\cdot,t}|\alpha)dJ_{i^{\mathcal{C}},t}d\delta_{\cdot,t} =$$
$$\frac{1}{\mathcal{Z}} \int \left\{ e^{-\alpha J_{i,t}^2 - 2\alpha\left(\sum_{j\neq i}|J'_{j,t}|\right)|J_{i,t}| - \alpha\left(\sum_{j\neq i}|J'_{j,t}|\right)^2} \times \right.$$
$$\left. \frac{1}{\mathcal{Z}_{i,t}}\left(\int e^{-\alpha {J'_{i,t}}^2 - 2\alpha\left(\sum_{j\neq i}|J'_{j,t}|\right)|J'_{i,t}|} dJ'_{i,t}\right)\right\} dJ'_{i^{\mathcal{C}},t}$$

Now, realizing that $\int e^{-\alpha {J'_{i,t}}^2 - 2\alpha\left(\sum_{j\neq i}|J'_{j,t}|\right)|J'_{i,t}|} dJ'_{i,t} = \mathcal{Z}_{i,t}$ and changing back $J'_{i^{\mathcal{C}},t}$ to $J_{i^{\mathcal{C}},t}$, we can easily obtain:

$$\int p(J_{\cdot,t},\delta_{\cdot,t}|\alpha)dJ_{i^{\mathcal{C}},t}d\delta_{\cdot,t} =$$
$$\int \frac{1}{\mathcal{Z}} e^{-\alpha\|J_{\cdot,t}\|_1^2} dJ_{i^{\mathcal{C}},t} = \int p(J_{\cdot,t}|\alpha)dJ_{i^{\mathcal{C}},t} \qquad \blacksquare$$

C. Parameters and hyperparameters posterior analysis

The following identity holds (Magnus and Neudecker, 2007):

$$N(V_{\cdot,t}|KJ_{\cdot,t},\beta_t I)N\left(J_{\cdot,t}\middle|0,diag(\Lambda_{\cdot,t})\right) =$$
$$= N(V_{\cdot,t}|K\mu_{\cdot,t},\beta_t I)N\left(\mu_{\cdot,t}\middle|0,diag(\Lambda_{\cdot,t})\right)|2\pi\bar{\Sigma}_t|^{\frac{1}{2}}N(J_{\cdot,t}|\mu_{\cdot,t},\bar{\Sigma}_t) \quad \text{[C1]}$$

where the posterior mean of parameters (maximum a posteriori estimate) is $\mu_{\cdot,t} = \frac{1}{\beta_t}\bar{\Sigma}_t K^{\mathcal{T}} V_{\cdot,t}$ and the posterior covariance matrix is $\bar{\Sigma}_t = \left(\frac{1}{\beta_t}K^{\mathcal{T}}K + \left(diag(\Lambda_{\cdot,t})\right)^{-1}\right)^{-1}$.

The posterior of hyperparameters can be obtained by substituting [F1] in [2-24] and integrating over J:

$$p(\Theta|V) \propto p(V,\Theta) = \int p(V,J,\Theta)dJ =$$
$$= \prod_t\left\{N(V_{\cdot,t}|K\mu_{\cdot,t},\beta_t I)N\left(\mu_{\cdot,t}\middle|0,diag(\Lambda_{\cdot,t})\right)|2\pi\bar{\Sigma}_t|^{\frac{1}{2}}p(\Theta_t)\right\} \quad \text{[C2]}$$

D. Update equations for ENET and ELASSO models

	**ENET**	
a)	$\hat{\mu}_{\cdot,t} = \frac{1}{\beta_t}\bar{\Sigma}_t K^{\mathcal{T}} V_{\cdot,t}$	[D1]
b)	$\widehat{\bar{\Lambda}}_{i,t} = \hat{\eta}_{i,t}/(k_t + \hat{\eta}_{i,t})$ $\hat{\eta}_{i,t} = -\frac{1}{4} + \sqrt{\frac{1}{16} + (\mu_{i,t}^2 + \bar{\Sigma}_{ii,t})\alpha_{1,t}k_t}$	[D2]
c)	$\hat{\alpha}_{1,t} = \left(\frac{S}{2}\right)/\sum_i\left\{\frac{(\mu_{i,t}^2+\bar{\Sigma}_{ii,t})}{\bar{\Lambda}_{i,t}}\right\}$	[D3]
	$\hat{k}_t = argmin|F(k_t)|$	[D4]
	$F(k_t) = \sum_i\left\{\frac{1}{1-\bar{\Lambda}_{i,t}}\right\} + \upsilon - \left(\tau - \frac{S}{2}\right)\frac{1}{k_t} -$ $-S\,(\pi k_t)^{-\frac{1}{2}}e^{-k_t}/\int_{k_t}^{\infty} Ga\left(x\middle|\frac{1}{2},1\right)dx$	[D5]
d)	$\hat{\beta}_t = \|V_{\cdot,t} - K\mu_{\cdot,t}\|_2^2/\left(N + \sum_i\left(\frac{2\alpha_{1,t}\bar{\Sigma}_{ii,t}}{\bar{\Lambda}_{i,t}}\right) - S\right)$	[D6]
	**ELASSO**	
a)	$\hat{\mu}_{\cdot,t} = \frac{1}{\beta_t}\bar{\Sigma}_t K^{\mathcal{T}} V_{\cdot,t}$	[D7]
b)	$\widehat{\bar{\Lambda}}_{i,t} = \hat{\eta}_{i,t}/(\alpha\delta_{i,t}^2 + \hat{\eta}_{i,t})$ $\hat{\eta}_{i,t} = -\frac{1}{4} + \sqrt{\frac{1}{16} + (\mu_{i,t}^2 + \bar{\Sigma}_{ii,t})\alpha^2\delta_{i,t}^2}$	[D8]
c)	$\hat{\alpha} = argmin|F(\alpha)|$	[D9]
	$F(\alpha) = \sum_{i,t}\left\{\frac{(\mu_{i,t}^2+\bar{\Sigma}_{ii,t})}{\bar{\Lambda}_{i,t}} + \frac{\delta_{i,t}^2}{1-\bar{\Lambda}_{i,t}}\right\} + \sum_t\|W^{-1}\delta_{\cdot,t}\|_1^2 -$ $-\frac{ST}{2\alpha} - \sum_{i,t}\left\{\frac{(\pi\alpha\delta_{i,t}^2)^{-\frac{1}{2}}e^{-\alpha\delta_{i,t}^2}\delta_{i,t}^2}{\int_{\alpha\delta_{i,t}^2}^{\infty} Ga\left(x|\frac{1}{2},1\right)dx}\right\}$	[D10]
	$\hat{\delta}_{i,t} = \sum_{j\neq i}|\hat{\mu}_{j,t}|$	[D11]
d)	$\hat{\beta}_t = \|V_{\cdot,t} - K\mu_{\cdot,t}\|_2^2/\left(N + \sum_i\left(\frac{2\alpha\bar{\Sigma}_{ii,t}}{\bar{\Lambda}_{i,t}}\right) - S\right)$	[D12]

Derivation of each update equation

a) Parameters. In both ENET and ELASSO we obtain:

$$\frac{\partial\mathcal{L}}{\partial\mu_{\cdot,t}} = \frac{\partial}{\partial\mu_{\cdot,t}}\frac{1}{2}\mu_{\cdot,t}^{\mathcal{T}}\left(diag(\Lambda_{\cdot,t})\right)^{-1}\mu_{\cdot,t} + \frac{\partial}{\partial\mu_{\cdot,t}}\frac{1}{2\beta_t}\|V_{\cdot,t} - K\mu_{\cdot,t}\|_2^2$$
$$= \left(diag(\Lambda_{\cdot,t})\right)^{-1}\mu_{\cdot,t} + \frac{1}{\beta_t}K^{\mathcal{T}}K\mu_{\cdot,t} - \frac{1}{\beta_t}K^{\mathcal{T}}V_{\cdot,t}$$
$$= -\frac{1}{\beta_t}K^{\mathcal{T}}V_{\cdot,t} + \left(\frac{1}{\beta_t}K^{\mathcal{T}}K + \left(diag(\Lambda_{\cdot,t})\right)^{-1}\right)\mu_{\cdot,t}$$

Using $\bar{\Sigma}_t = \left(\frac{1}{\beta_t}K^{\mathcal{T}}K + \left(diag(\Lambda_{\cdot,t})\right)^{-1}\right)^{-1}$ and equating to zero we obtain:

$$\hat{\mu}_{\cdot,t} = \frac{1}{\beta_t}\bar{\Sigma}_t K^{\mathcal{T}} V_{\cdot,t} \qquad \blacksquare$$

b) Hyperparameters. For ENET we will have:

$$\frac{\partial\mathcal{L}}{\partial\bar{\Lambda}_{i,t}} = \frac{\partial}{\partial\bar{\Lambda}_{i,t}}\frac{1}{2}\log|\bar{\Sigma}_t^{-1}| + \frac{\partial}{\partial\bar{\Lambda}_{i,t}}\frac{1}{2}\log|diag(\Lambda_{\cdot,t})| +$$
$$+ \frac{\partial}{\partial\bar{\Lambda}_{i,t}}\frac{1}{2}\mu_{\cdot,t}^{\mathcal{T}}\left(diag(\Lambda_{\cdot,t})\right)^{-1}\mu_{\cdot,t} - \frac{\partial}{\partial\bar{\Lambda}_{i,t}}\log Ga\left(\gamma_{i,t}\middle|\frac{1}{2},1\right)$$

Deriving and reorganizing terms:

$$\frac{\partial \mathcal{L}}{\partial \overline{\Lambda}_{i,t}} = -\left(\bar{\Sigma}_{ii,t} + \mu_{i,t}^2\right)\frac{\alpha_{1,t}}{\overline{\Lambda}_{i,t}^2} + \frac{1}{2\overline{\Lambda}_{i,t}} + \frac{1}{2\left(1-\overline{\Lambda}_{i,t}\right)} + \frac{k_t}{\left(1-\overline{\Lambda}_{i,t}\right)^2}$$

Where $\bar{\Sigma}_{ii,t}$ is the i-th diagonal element of $\bar{\Sigma}_t$. Equating to zero and using the change of variable $\overline{\Lambda}_{i,t} = \eta_{i,t}/\left(\eta_{i,t} + k_t\right)$ we obtain the equation:

$$-\left(\bar{\Sigma}_{ii,t} + \mu_{i,t}^2\right)\alpha_{1,t}k_t^2 + \frac{k_t\eta_{i,t}}{2} + k_t\eta_{i,t}^2 = 0$$

Where the only positive root is:

$$\hat{\eta}_{i,t} = -\frac{1}{4} + \sqrt{\frac{1}{16} + \left(\mu_{i,t}^2 + \bar{\Sigma}_{ii,t}\right)\alpha_{1,t}k_t}$$

So that if we define $\hat{\gamma}_{i,t} = \hat{\eta}_{i,t} + k_t$ we will have:

$$\overline{\Lambda}_{i,t} = \hat{\eta}_{i,t}/\hat{\gamma}_{i,t}$$

For the ELASSO model we follow the same procedure with respective change of variables $\alpha_{1,t} = \alpha$ and $k_t = \alpha\delta_{i,t}^2$ ■

c) Hyperparameters. For ENET we will have:

$$\frac{\partial \mathcal{L}}{\partial \alpha_{1,t}} = \frac{\partial}{\partial \alpha_{1,t}}\left\{\frac{1}{2}\log\left|\bar{\Sigma}_t^{-1}\right| + \frac{1}{2}\log\left|diag\left(\Lambda_{\cdot,t}\right)\right| + \frac{1}{2}\mu_{\cdot,t}^{\mathcal{T}}\left(diag\left(\Lambda_{\cdot,t}\right)\right)^{-1}\mu_{\cdot,t}\right\}$$

Substituting $\Lambda_{i,t} = \overline{\Lambda}_{i,t}/\alpha_{1,t}$ and equating to zero:

$$\frac{\partial \mathcal{L}}{\partial \alpha_{1,t}} = \sum_i \frac{\bar{\Sigma}_{ii,t}+\mu_{i,t}^2}{\overline{\Lambda}_{i,t}} + \sum_i \frac{1}{2}\frac{\partial}{\partial \alpha_{1,t}}\log\frac{1}{\alpha_{1,t}}$$

$$\alpha_{1,t} = (S/2)/\sum_i\left\{\frac{\bar{\Sigma}_{ii,t}+\mu_{i,t}^2}{\overline{\Lambda}_{i,t}}\right\}$$

For the hyperparameter defined by [2-5] we will have:

$$\frac{\partial \mathcal{L}}{\partial k_t} = \frac{\partial}{\partial k_t}\left\{-\sum_i \log Ga\left(\gamma_{i,t}\middle|\frac{1}{2},1\right) + \sum_i \log\int_{k_t}^{\infty} Ga\left(x\middle|\frac{1}{2},1\right)dx - \log p(k_t)\right\}$$

Substituting $\gamma_{i,t} = k_t/\left(1-\overline{\Lambda}_{i,t}\right)$:

$$\frac{\partial \mathcal{L}}{\partial k_t} = \sum_i \frac{1}{1-\overline{\Lambda}_{i,t}} - S\frac{Ga\left(k_t\middle|\frac{1}{2},1\right)}{\int_{k_t}^{\infty} Ga\left(x\middle|\frac{1}{2},1\right)dx} + \upsilon - \left(\tau - \frac{S}{2}\right)\frac{1}{k_t}$$

For the ELASSO model we have:

$$\frac{\partial \mathcal{L}}{\partial \alpha} = \frac{\partial}{\partial \alpha}\sum_t\left\{\frac{1}{2}\log\left|\bar{\Sigma}_t^{-1}\right| + \frac{1}{2}\log\left|diag\left(\Lambda_{\cdot,t}\right)\right| + \frac{1}{2}\mu_{\cdot,t}^{\mathcal{T}}\left(diag\left(\Lambda_{\cdot,t}\right)\right)^{-1}\mu_{\cdot,t} - \sum_i \log Ga\left(\gamma_{i,t}\middle|\frac{1}{2},1\right) + \sum_i \log\int_{\alpha\delta_{i,t}^2}^{\infty} Ga\left(x\middle|\frac{1}{2},1\right)dx - \frac{S}{2}\log\alpha + \alpha\left\|W^{-1}\delta_{\cdot,t}\right\|_1^2\right\}$$

$$= \sum_{it}\frac{1}{2}\bar{\Sigma}_{ii,t}\frac{\partial}{\partial\alpha}\frac{1}{\Lambda_{i,t}} + \sum_{it}\frac{1}{2}\frac{\partial}{\partial\alpha}\log\Lambda_{i,t} + \sum_{it}\frac{1}{2}\mu_{i,t}^2\frac{\partial}{\partial\alpha}\frac{1}{\Lambda_{i,t}} + \sum_{it}\frac{1}{2}\frac{\partial}{\partial\alpha}\log\alpha + \sum_{it}\frac{\delta_{i,t}^2}{1-\overline{\Lambda}_{i,t}} - \sum_{it}\frac{\delta_{i,t}^2 Ga\left(\alpha\delta_{i,t}^2\middle|\frac{1}{2},1\right)}{\int_{\alpha\delta_{i,t}^2}^{\infty} Ga\left(x\middle|\frac{1}{2},1\right)dx} - \frac{ST}{2}\frac{1}{\alpha} + \sum_t\left\|W^{-1}\delta_{\cdot,t}\right\|_1^2$$

Substituting $\Lambda_{i,t} = \overline{\Lambda}_{i,t}/\alpha$ and $\gamma_{i,t} = \alpha\delta_{i,t}^2/\left(1-\overline{\Lambda}_{i,t}\right)$:

$$\frac{\partial \mathcal{L}}{\partial \alpha} = \sum_{it}\frac{\bar{\Sigma}_{ii,t}+\mu_{i,t}^2}{\overline{\Lambda}_{i,t}} + \sum_{it}\frac{\delta_{i,t}^2}{1-\overline{\Lambda}_{i,t}} + \sum_t\left\|W^{-1}\delta_{\cdot,t}\right\|_1^2 - \frac{ST}{2}\frac{1}{\alpha} - \sum_{it}\frac{\delta_{i,t}^2 Ga\left(\alpha\delta_{i,t}^2\middle|\frac{1}{2},1\right)}{\int_{\alpha\delta_{i,t}^2}^{\infty} Ga\left(x\middle|\frac{1}{2},1\right)dx}$$ ■

d) Hyperparameters. In both ENET and ELASSO we will have:

$$\frac{\partial \mathcal{L}}{\partial \beta_t} = \frac{\partial}{\partial \beta_t}\frac{1}{2}\log\left|\bar{\Sigma}_t^{-1}\right| + \frac{\partial}{\partial \beta_t}\frac{1}{2}\log\left|\beta_t I\right| + \frac{\partial}{\partial \beta_t}\frac{1}{2\beta_t}\left\|V_{\cdot,t} - K\mu_{\cdot,t}\right\|_2^2$$

$$= \frac{1}{2\beta_t}\left[tr\left(\frac{\bar{\Sigma}_t}{diag\left(\Lambda_{\cdot,t}\right)}\right) - S\right] + \frac{N}{2}\frac{1}{\beta_t} - \frac{1}{2{\beta_t}^2}\left\|V_{\cdot,t} - K\mu_{\cdot,t}\right\|_2^2$$

Equating to zero we obtain:

$$\beta_t = \frac{\left\|V_{\cdot,t} - K\mu_{\cdot,t}\right\|_2^2}{N + \sum_{i=1}^{S}\left(\frac{\bar{\Sigma}_{ii,t}}{\Lambda_{i,t}}\right) - S}$$

From where it is easy to get equations [D6] and [D12] by using [2-4] and [2-20] for ENET and ELASSO, respectively. ■

E. Inference strategy and implementation details

The high computational cost for obtaining $\bar{\Sigma}_t$ by means of a matrix inversion operation can be avoided by using the economical singular value decomposition (SVD) of the lead field $K = LDR^{\mathcal{T}}$, and the Woodbury identity (Magnus and Neudecker, 2007), leading to:

$$\bar{\Sigma}_t = \beta_t diag\left(\Lambda_{\cdot,t}\right)R\left(R^{\mathcal{T}}diag\left(\Lambda_{\cdot,t}\right)R + \beta_t D^{-2}\right)^{-1}D^{-2}R^{\mathcal{T}} \quad [2\text{-}26]$$

The update formulas in Proposition 2.3.2 a), b) are consistent with the sparsity constraint in both the ENET and ELASSO models, since the elements of the effective prior variance matrix Λ (or equivalently $\overline{\Lambda}$) select which elements of μ become zero. When $\Lambda_{i,t} \to 0$, the i-th row and i-th -column of the matrix $\bar{\Sigma}_t$ in [2-26] tend to zero vectors, from where $\hat{\mu}_{i,t} \to 0$. In the same way, if some parameters are very small in a previous iteration ($\hat{\mu}_{i,t} \approx 0$, i-th diagonal element $\bar{\Sigma}_{ii,t} \approx 0$), they will lead to $\Lambda_{i,t} \to 0$ in the next iteration (equations [D2] and [D8]). In some algorithms, this property usually means that if one activation is set to zero (e.g. removed from the active set) in an iteration, it will not appear as part of the solution. In our case, however, we do not prune to zero the small coefficients. Therefore, although unlikely, a "zeroed" activation might be re-estimated in a future iteration and contribute to the solution.

The non-linear terms in [D5] and [D10] are obtained from the derivative of the normalization constants in [2-8] and [2-21]. These terms decrease strictly with respect to their arguments leading to smaller values of F for higher values of k_t and α, which is equivalent in both cases to have more zero elements in $\overline{\Lambda}$. The measurements variance β_t in [D6] and [D12] is generally considered superfluous in the learning process, because it only acts as a scale factor for the parameters and usually decelerates the algorithm convergence (Babacan et al., 2010). In our case, we fix it to β_t=1, for all time points.

We also use fixed values for the parameters of the Gamma distribution [2-11] in the ENET model. In particular we chose τ=S, which preserves the monotony of [D5] (in the sense that only one zero of F exists), and υ=ϵS, where ϵ is such that k_t has a flexible prior, with mean(k_t)≈ $1/\epsilon$ and variance(k_t)≈ $1/(\epsilon^2 S)$. Obviously, the value of k_t that optimizes $\mathcal{L}$ will depend on (τ, υ), since these have some influence in the intercept of [D5]. In order to keep an adequate balance we impose identical prior to α_1, which allows the flexibility in our learning of different degrees of sparsity. Although optimal values for (τ, υ) might also be estimated within the Empirical Bayes (setting their corresponding priors), we only consider here an exploratory study where they are fixed in the ENET model.

F. Pseudo code for the algorithms

Algorithm ENET-SSBL

INPUT: $\boldsymbol{K}$, $\boldsymbol{V}$
OUTPUT: $\boldsymbol{\mu}$, $\boldsymbol{\beta}$, $\boldsymbol{\alpha_1}$, $\boldsymbol{k}$
For all $\boldsymbol{t} = \overline{\boldsymbol{1}, \boldsymbol{T}}$.
  Initialize $\boldsymbol{\Lambda}_{\cdot,t}$.
  Iterate until convergence criteria holds
    Compute $\boldsymbol{\bar{\Sigma}_t}$ [2-26].
    Update $\boldsymbol{\mu}_{\cdot,t}$ [D1], $\boldsymbol{\bar{\Lambda}}_{\cdot,t}$ [D2], $\boldsymbol{\alpha_{1,t}}$ [D3], $\boldsymbol{k_t}$ [D4]
    and $\boldsymbol{\beta_t}$ [D6].
  End
End

Algorithm ELASSO-SSBL

INPUT: $\boldsymbol{K}$, $\boldsymbol{V}$
OUTPUT: $\boldsymbol{\mu}$, $\boldsymbol{\beta}$, $\boldsymbol{\alpha}$
Initialize $\boldsymbol{\Lambda}$.
Iterate until convergence criteria holds
  For all $\boldsymbol{t} = \overline{\boldsymbol{1}, \boldsymbol{T}}$.
    Compute $\boldsymbol{\bar{\Sigma}_t}$ [2-26].
    Update $\boldsymbol{\mu}_{\cdot,t}$ [D7], $\boldsymbol{\bar{\Lambda}}_{\cdot,t}$ [D8], $\boldsymbol{\delta}_{\cdot,t}$ [D11]
    and $\boldsymbol{\beta_t}$ [D12].
  End
Update $\boldsymbol{\alpha}$ [D9].
End

G. Mathematical notation

Symbol	Description
$\mathcal{V}(\cdot)$	Continuous function that represents the scalp voltage, dependent on scalp coordinates (r_e) and time (t).
r_e	Scalp coordinates.
V	NxT spatio-temporal matrix that represents the scalp voltage (data), rows represent sensors and columns represent time points.
N	Number of scalp sensors.
ε	NxT spatio-temporal matrix that represents sensors' noise.
$\mathcal{J}(\cdot)$	Continuous function that represents the PCD, dependent on the source's space coordinates (r) and time (t).
r	Sources' space coordinates.
$\boldsymbol{dr^3}$	Volumetric differential element in the sources' space.
J	SxT spatio-temporal matrix that represents the PCD (parameters), rows represent points within the discretized sources' space and columns represent time points.
S	Number of points in the discrete sources' space
i, j	Indexes used to represent points within the discretized sources' space.
$J_{\cdot,t}$	t'th column vector of the spatio-temporal parameters matrix (PCD).
$J_{i^c,t}$	$S-1$ dimensional column vector obtained from $J_{\cdot,t}$ by subtracting the i'th element
$dJ_{i^c,t}$	$S-1$ dimensional volumetric differential element of the $J_{i^c,t}$ column vector.
$\mathcal{K}(\cdot)$	Continuous function that represents the Lead Field, dependent on scalp coordinates (r_e) and source space coordinates (r).
K	NxS Lead Field matrix.
$\mathbb{L}$	SxS matrix that represents the Laplacian operator.
t	Continuous/discrete time index.
T	Number of time points.
$P(\cdot)$	Function that represents the constraints or penalties.
λ	Regularization parameter.
$p(\cdot)$	Probability density function
$\mathcal{Z}$	Normalization constant of some of the probability density functions
α,α_1,α_2	Different model's hyperparameters (precisions).
β	Noise variance's hyperparameter.
k	Lower truncation limit (hyperparameter) of the Truncated Gamma distribution.
γ	SxT matrix of the new hyperparameters derived from scaled Gaussian mixtures procedures.
δ	SxT matrix of the new hyperparameters derived from the Elitist Lasso hierarchization.
$\bar{\mathcal{Z}}$	Normalization constant of the hyperparameter δ probability density function.
Λ	SxT matrix of parameter's variances in the hierarchical ENet and ELasso models.
$\bar{\Lambda}$	SxT matrix proportional to parameter's variances in the hierarchical Elastic Net and Elitist Lasso models.
τ, υ	Scale and shape of the hyperparameter's Gamma prior.
x	General variable used to represent Markov Random Fields and some integrals.
$x_{\mathcal{H}}$, $x_{\mathcal{H}^c}$	General variable that furnishes a subset of elements indexed by $\mathcal{H}$ within the vector x and its complement correspondingly.
$\mathcal{P}(\cdot)$, $\mathcal{P}_{ij}(\cdot)$	Potentials of the Elitist Lasso spatial Markov Random Field.
$\mathfrak{I}$	General variable that represent a set of indexes.
$1_{S\times S}$	SxS matrix of ones.
$\mathbb{I}_{S\times S}$	SxS identity matrix
$I_{\mathcal{R}_+^S}$	Indicator function of the set of non-negative coordinates points in the S dimensional real space.
$\Delta(\cdot)$	Dirac delta distribution.
Θ, Θ_t	Variables that correspondingly embrace all hyperparameters of the model and all hyperparameters of the model for a single time point.
μ	Variable that represents the mean of the parameters Gaussian posterior distribution.
$\bar{\Sigma}$	Parameter's posterior distribution covariance matrix.
